# Acute uncomplicated urinary tract infections and subsequent type 2 diabetes diagnosis in women: a national cohort study including primary healthcare data

**DOI:** 10.1080/02813432.2025.2580905

**Published:** 2025-11-06

**Authors:** Filip Jansåker, Xinjun Li, Ola Ekström, Henning Stenberg, Kristina Sundquist

**Affiliations:** a Department of Clinical Sciences, Malmö, Center for Primary Health Care Research, Lund University, Malmö, Sweden; b University Clinic Primary Care, Skåne University Hospital, Malmö, Sweden; c Department of Clinical Microbiology, Center of Diagnostic Investigations, Rigshospitalet, Copenhagen, Denmark; d Department of Clinical Sciences, Genomics, Diabetes and Endocrinology Unit, Lund University Diabetes Center, Lund University, Malmö, Sweden; e Department of Functional Pathology, School of Medicine, Center for Community-based Healthcare Research and Education (CoHRE), Shimane University, Matsue, Japan

**Keywords:** Cystitis, diagnosis, pyelonephritis, type 2 diabetes, UTI, women

## Abstract

*Purpose*: To examine the association between uncomplicated urinary tract infections and subsequent type 2 diabetes (T2D) diagnosis in women. *Materials and methods*: We included 1,840,044 women without previously diagnosed type 2 diabetes (T2D) or redeemed antidiabetic drugs. *Results and conclusions*: During the 12-year study period, women with uncomplicated urinary tract infections (cystitis and pyelonephritis), diagnosed within two years before T2D, did not have higher subsequent risks of T2D compared to those without urinary tract infections.

## Introduction

In Sweden, a country of universal healthcare, the estimated proportion of undiagnosed T2D is 33% [1]. Globally, nearly half of all type 2 diabetes (T2D) cases may be undiagnosed [2]. Identifying clinical indicators of T2D could help to identify undiagnosed cases of T2D.

Infections diagnosed in primary healthcare are common in patients with T2D [3] and it is possible that common infections could be useful predictors of T2D. Research on this topic is, however, sparse. Previous research has shown that patients with community-acquired pneumonia have high prevalence of prediabetes and undiagnosed diabetes [4]. In addition, women with vulvovaginal candidiasis have increased subsequent risk of T2D [5]. It is also well-established that women with T2D have a higher risk of subsequent UTI compared to women without T2D [3,6–10]. There are several possible explanations behind the increased risk of UTI in women with T2D; for example, T2D is associated with increased concentration of glucose in the urine- and renal parenchyma as well as impaired bladder function and weakened immune system [7–10]. Thus, it is plausible that UTI also could be a predictor of a subsequent risk of T2D. Such knowledge could be useful in clinical practice as UTI is a common infection in women worldwide [11].

The purpose of this nationwide study, including primary healthcare data, was to examine the association between uncomplicated UTI and subsequent T2D diagnosis in women without previously diagnosed diabetes mellitus. With an aim to evaluate whether such an association could provide useful information for clinical practice, the analysis was restricted to UTIs occurring within two years before T2D diagnosis [5].

## Methods

### Design and population

A nationwide open cohort study of 1,840,044 women aged 35–65 years (2007–2018) was conducted. We included women aged 35–65 years as the prevalence of T2D and risk factors (e.g. urological and other structural abnormalities) for UTI are lower in the younger age groups [1,2,11]. We excluded 46,049 women with either a diagnosis of T2D (ICD-10, code ‘E11’) or redeemed anti-diabetic medicine (ATC code ‘A10’, i.e. ‘Drugs used in diabetes’, including insulin), within two years (2005–2006) prior to the study period.

### Data sources

Population-based (almost nationwide) primary healthcare data (1997–2018) from 20 out of 21 administrative regions in Sweden were used. The coverage of these data varied by time and region based on when patient records became digitalized; the coverage was around 72% of the Swedish population in 2015 and around 90% at the end of the study. The following national registers were also used: the Swedish Prescribed Drug Register (2005–2018), including data on redeemed prescriptions in Sweden; the Medical Birth Register (1973–2018); and the National Patient Register (NPR), including data on inpatient (1964–2018) and outpatient care (2001–2018); the Total Population Register (1968–2018), containing data on death, emigration and sociodemographic factors, and the Multi-Generation Register (1932–2018), containing data on (biological) family lineages. All linkages between the individual-level data in the datasets were performed using a pseudonymized version of the unique Swedish 10-digit personal identification number.

### Variables

The outcome was T2D, measured as ICD-10 code ‘E11’. The potential predictors were uncomplicated cystitis (ICD-10, ‘N30’) and pyelonephritis (ICD-10, ‘N10’ or ‘N12’). T2D and UTIs were identified in primary healthcare data and NPR. For cystitis, the specific ICD-10 codes of N301–304, and N308 were not included as these codes do not represent acute cystitis. We did not consider UTI events as potential predictors when obvious risk factors for complicated UTI [11,12] were present at the time of the UTI diagnosis, such as ongoing pregnancy or treatment with immunomodulating agents, or previously diagnosed immunodeficiency disorders or urological abnormalities (ICD-10: B20–24, C64–68, D41, D80–89, M623, N03, N07, N11, N13–23, N25–29, N32 and Q60–64). Covariates such as age and other sociodemographic factors, parity and family history of T2D were included to adjust for potential confounders.

### Statistical analysis

The study period started 1 January 2007, and follow-up proceeded until diagnosis of T2D, death, emigration or end of the study (31 December 2018), whichever came first. Cox regression models were used to estimate hazard ratios (HRs) and 95% confidence intervals (CIs) for T2D in women with a UTI compared to women without UTI. To examine whether UTI could be a useful predictor of T2D, we measured UTI episodes (first event) that occurred within two years prior to a T2D diagnosis during the study period. The analyses were conducted for cystitis and pyelonephritis separately. A two-tailed *p* value of <0.05 was considered for statistical significance and SAS version 9.4 (SAS Institute Inc., Cary, NC) was used for all statistical analyses.

## Results

A total of 47,335 women were diagnosed with T2D during the study period corresponding to an incidence rate of 31.43 (95% CI, 31.14–31.71) per 10,000 person-years. Table 1 shows that women with a cystitis or a pyelonephritis diagnosis within two years prior to the T2D diagnosis had a lower risk of T2D compared to those without UTI. In women diagnosed with both UTI and T2D during follow-up, 70.5% of the women with cystitis and 57.4% of the women with pyelonephritis had a UTI within two years before the T2D diagnosis. For these women, the mean time to the T2D diagnosis was 0.64 years for cystitis and 0.62 years for pyelonephritis.

**Table 1.** The temporal association between urinary tract infections (cystitis and pyelonephritis) and incident type 2 diabetes in women aged 35–65 years during the study period 2007–2018 (*N* = 1,840,044).

Type of UTI	Women without UTI (%)^a^	Women without UTI with T2D (%)^b^	Women with UTI (%)^a^	Women with UTI and T2D during the study period (%)^b^	Women with UTI and subsequent T2D (%)^c^	HR^d^ (95% CI)	Mean follow-up time (range), years
Cystitis	1,645,547 (89.4)	41,617 (2.5)	194,497 (10.6)	5718 (2.9)	4033 (70.5)	0.84 (0.81–0.87)	0.64 (0–2)
Pyelonephritis	1,831,043 (99.5)	47,025 (2.6)	9001 (0.5)	310 (3.4)	178 (57.4)	0.77 (0.67–0.89)	0.62 (0–2)

CI: confidence interval; HR: hazard ratio; T2D: type 2 diabetes; UTI: urinary tract infection.

^a^ Proportion of study population without/with UTI diagnosis during the study period.

^b^ Proportion of those diagnosed without/with UTI who were diagnosed with T2D during the study period.

^c^ Proportion of those diagnosed with UTI and T2D during the study period who were diagnosed with UTI within two years before T2D diagnosis.

^d^ HR for subsequent T2D in those with UTI (diagnosed within two years before T2D) compared to those without UTI during the study period. A Cox regression model adjusted for age, family history of type 2 diabetes, parity and individual-level sociodemographic factors (education level, income level, region of residence, and country of origin).

## Discussion

In this nationwide population of 1,840,044 women aged 35–65 years, uncomplicated cystitis and pyelonephritis diagnosed within two years before T2D diagnosis were not associated with higher T2D risks compared to women without the infection. These findings suggest that uncomplicated UTIs may not be useful clinical predictors for T2D in this population and setting.

In contrast to these results, our previous findings on vulvovaginal candidiasis, diagnosed within two years prior to T2D diagnosis, indicated a higher subsequent risk of T2D in women aged ≥35 years [5]. Moreover, patients with community-acquired pneumonia have been found to have high prevalence of undiagnosed diabetes [4]. It is possible that the broad causes and high prevalence of UTI might dilute the predictive power of UTI as a clinical marker for T2D compared to other infections, at least in the general population of women presenting with symptoms of uncomplicated UTI in primary healthcare. However, more complicated and more severe forms of UTI (e.g. hospitalization for pyelonephritis) may be more strongly associated with undiagnosed T2D. Furthermore, while focusing on whether UTIs occurring within two years before the T2D diagnosis has clinical relevance, such an approach may constrain the potential associations between UTI and subsequent T2D. Including UTIs from longer time periods could thus show different results, which can be explored in future research into the association between UTI and subsequent T2D.

Although we did not identify predictive relationships between UTI and T2D, the sequence of UTI followed by T2D diagnosis within two years after the infections was observed in a majority of women diagnosed with both conditions during the 12-year study period may reflect differences in individual health trajectories and pathophysiological mechanisms of UTI in women with T2D [3,6–10]. Further studies could explore specific subgroups in which UTIs might serve as predictors for T2D, e.g. recurrent [13] and complicated UTI [12]. Future research could also consider the heterogeneity of diabetes as the association with UTI might differ across subtypes of diabetes [14]. For example, insulin resistance is linked to increased susceptibility to UTI [15], whereas high BMI has been associated with lower UTI risk in women [16]. It is also possible that UTI might be more valuable in the prediction of T2D in countries with higher prevalence of undiagnosed T2D. Another important aspect to consider when interpreting the findings from this study is that the outcome was T2D diagnosis. Given that the estimated proportion of undiagnosed T2D in Sweden is approximately 33%, and even higher for prediabetes [1], the ‘true’ association between UTI and undiagnosed T2D and prediabetes may be different than the observed association in this study, which warrants further studies. Future studies could, for example, examine the prevalence of T2D and prediabetes in women diagnosed with uncomplicated UTI, as previously done for patients with pneumonia [4].

The main limitation with our study is the lack of detailed clinical and microbiological data. Although other researchers have found significant bacteriuria in 80% of clinically diagnosed cases of uncomplicated cystitis (*n* = 304) in Swedish primary healthcare [17], we could not validate the UTI diagnoses in this nationwide study. Additionally, missed diagnoses of uncomplicated cystitis are possible [18], as some women may not seek medical attention (e.g. if symptoms are mild) and the infection is self-limiting in over 25% of cases [19]. However, any potential misclassification bias is probably lower for pyelonephritis, a more severe infection, and the associations were similar for cystitis and pyelonephritis. The major strength of this study is that we were able to use prospectively collected data from almost nationwide primary healthcare settings, where most UTI are diagnosed and managed [11,18]. Another strength is the highly complete national healthcare and population-based registers. For example, diagnoses in the National Patient Register are >99% complete and have a positive predictive value for diabetes mellitus of almost 99%. Together with the almost nationwide primary healthcare data, these data sources include more or less all women diagnosed with UTI and T2D during the study period. Altogether, this supports that the data sources used in this study are representative. Lastly, our data allowed us to restrict the analysis to women without previously diagnosed diabetes mellitus or redeemed antidiabetic drugs and other obvious risk factors for UTI, enabling a more accurate assessment of whether uncomplicated UTI could be an independent predictor for T2D in women.

In conclusion, in this nationwide study of women aged 35–65 years, uncomplicated UTI diagnosed within two years before T2D diagnosis did not predict a higher risk of T2D compared to women without UTI, at least not in primary healthcare settings. Further studies could explore specific subgroups in which UTIs might serve as predictors for T2D.

## Supplementary Material

Supplemental Material

Supplemental Material

Supplemental Material

## Data Availability

This study made use of several national registers and, owing to legal concerns, data cannot be made openly available. Further information regarding the health registries is available from the Swedish National Board of Health and Welfare: https://www.socialstyrelsen.se/en/statistics-and-data/registers/ (registerservice@socialstyrelsen.se) and Statistics Sweden: https://www.scb.se/en/services/ordering-data-and-statistics/ (scb@scb.se). The statistical code can be provided on reasonable request to the corresponding author (filip.jansaker@med.lu.se).
