# Supplementary material for: Acute uncomplicated urinary tract infections and subsequent type 2 diabetes diagnosis in women: a national cohort study including primary healthcare data
Source: Scand J Prim Health Care. 2025 Nov 6;44(1):1–5. doi: 10.1080/02813432.2025.2580905 (PMC12918306; doi:10.1080/02813432.2025.2580905)
Supplement: Supplemental Material [file IPRI_A_2580905_SM7509.docx]

**Supplemental data**

**Dose-response assessment of neuroactive botanical extracts and their bioactive constituents using microelectrode array (MEA) recordings in rat primary cortical cultures**

Regina G.D.M. van Kleef^1^, J. Pepijn Wopken^1^, Julie Krzykwa^2^, Constance A. Mitchell^2^, Remco H.S. Westerink^1,*^

**Author affiliations**

^1^ Neurotoxicology Research Group, Division of Toxicology, Institute for Risk Assessment Sciences (IRAS), Faculty of Veterinary Medicine, Utrecht University, P.O. Box 80.177, NL-3508 TD Utrecht, The Netherlands

^2^ Health and Environmental Sciences Institute, Washington, DC, USA

^*^ Correspondence and reprint requests to R.H.S. Westerink, Neurotoxicology Research Group, Division of Toxicology, Institute for Risk Assessment Sciences (IRAS), Faculty of Veterinary Medicine, Utrecht University, P.O. Box 80.177, NL-3508 TD Utrecht, The Netherlands; E-mail: R.Westerink@uu.nl

**Supplementary Table S1. List of neuroactive botanical extracts used in this study**, including their standardized common and scientific names, Distributed Structure-Searchable Toxicity (DSSTox) substance identifier (DTXSID), and the part(s) of the plant used to derive the botanical extract. Botanicals with suspected neuroactive potential are in bold (from Kanungo et al., 2024).

| **Standardized Common Name** | **Scientific Name** | **DTXSID** | **Plant part(s)** | **Details** |
| --- | --- | --- | --- | --- |
| **Aconite** | ***Aconitum napellus* L.** | DTXSID701061676 | Whole plant | 95% ethanol extract |
| Aristolochia fangchi | *Aristolochia fangchi* Y.C. Wu ex L.D. Chou & S.M. Hwang | DTXSID201349132 | Root | 95% ethanol extract |
| Blue cohosh | *Caulophyllum thalictroides* (L.) Michx. | DTXSID401042859 | Root & Rhizome | 95% ethanol extract |
| Ephedra | *Ephedra sinica* Stapf | DTXSID801018482 | Aerial Parts | 95% ethanol extract |
| Green tea | *Camellia sinensis* (L.) Kuntze | DTXSID0031398 | Leaf | Green tea dry decaffeinated extract;  78.5% total catechins (54.6% (-)-epigallocatechin-3-O-gallate) |
| Goldenseal | *Hydrastis canadensis* L. | DTXSID40274228 | Root & Rhizome | 95% ethanol extract |
| **Kava** | ***Piper methysticum* G. Forst.** | DTXSID901018742 | Root & Rhizome | 95% ethanol extract |
| **Kratom** | ***Mitragyna speciosa* Korth.** | DTXSID001334842 | Leaf | 95% ethanol extract |
| Milk thistle | *Silybum marianum* (L.) Gaertn. | DTXSID8031657 | Seed | Milk thistle dry extract; 90.6% of silymarin isomers calc. as silibinin |
| **Oleander** | ***Nerium oleander* L.** | DTXSID201042091 | Leaf | 95% ethanol extract |
| Usnea | *Usnea* spp. | DTXSID701349537 | Whole Lichen | 95% ethanol extract |
| **Tripterygium^[[1]](#footnote-1)^** | ***Tripterygium wilfordii* Hook. f.** | DTXSID301349830 | Root | 95% ethanol extract |
| **Yohimbe** | ***Corynanthe johimbe*K.Schum., syn. *Pausinystalia johimbe* (K. Schum.) Pierre ex Beille** | DTXSID4032291 | Bark | 95% ethanol extract |

**Supplementary Table S2**. **List of neuroactive botanical extracts and their active constituents at the concentrations that they are present in the extracts**, including their standardized common and scientific names, Distributed Structure-Searchable Toxicity (DSSTox) substance identifier (DTXSID), molecular weight of the constituent and the concentrations of the constituent in the botanical extract at the dose levels indicated.

| **Plant name** | **Scientific name** | **Constituent** | **Constituent DTXID** | **MW (g/mol)** | **Measured Concentration (mg/g extract)** | **[Constituent] in 50 μg/mL (μM)** | **[Constituent] in 25 μg/mL (μM)** | **[Constituent] in 5 μg/mL (μM)** | **[Constituent] in 1 μg/mL (μM)** |
| --- | --- | --- | --- | --- | --- | --- | --- | --- | --- |
| Kava | *Piper methysticum* | dihydrokavain | DTXSID 101018162 | 232,3 | 112,5 | 24,2 | 12,11 | 2,42 | 0,48 |
| Goldenseal | *Hydrastis canadensis* | Berberine | DTXSID 9043857 | 336,4 | 8,12 | 1,2 | 0,60 | 0,12 | 0,02 |
| Green tea | *Camellia sinensis* | (−)-Epigallocatechin-3-O-gallate | DTXSID 1029889 | 458,4 | 440 | 48 | 24 | 4,80 | 0,96 |
| Kratom | *Mitragyna speciosa* | Mitragynine | DTXSID 701032140 | 398,5 | 60,09 | 7,5 | 3,77 | 0,75 | 0,15 |
| Milk thistle | *Silybum marianum* | Silybin-B | DTXSID 30858697 | 482,4 | 239,3 | 24,8 | 12,4 | 2,48 | 0,50 |
| Yohimbe | *Corynanthe johimbe* | Yohimbine Hydrochloride | DTXSID 9040130 | 354,4 | 49,5 | 6,98 | 3,49 | 0,70 | 0,14 |
| Oleander | *Nerium oleander* | Oleandrin | DTXSID 40861950 | 576,7 | 7,611 | 0,66 | 0,33 | 0,066 | 0,013 |
| Aconite | *Aconitum napellus*. | Aconitine | DTXSID 4046319 | 645,7 | 0,972 | 0,075 | 0,038 | 0,008 | 0,002 |

**Supplementary Table S3**. Set of the eight most important micro-electrode array (MEA) parameters.

| Category | Metrics Parameters | Description |
| --- | --- | --- |
| Spike parameters | Number of spikes | Total number of spikes over the duration of the analysis |
|  | | |
| Burst  parameters | Number of bursts | Total number of bursts (cluster of spikes measured on a single electrode) over the duration of the analysis |
|  | Burst duration | Average time from the first spike in a burst till the last spike (s). Longer bursts indicate more excitation as it takes longer to shut down a burst |
|  | Number of spikes per burst | Average number of spikes occurring in a burst |
|  | | |
| Network burst  parameters | Number of network bursts | Total number of network bursts (coordinated cluster of spiking across multiple electrodes) over the duration of the analysis |
|  | Network burst duration | Average time from the first spike till the last spike in a network burst (s). Longer bursts indicate more excitation as it takes longer to shut down a burst |
|  | Number of spikes per network burst | Average number of spikes occurring in a network burst |
|  | | |
| Synchronicity parameters | Area under cross-correlation | Area under inter-electrode cross-correlation. The higher the value, the greater the synchronicity of the network |


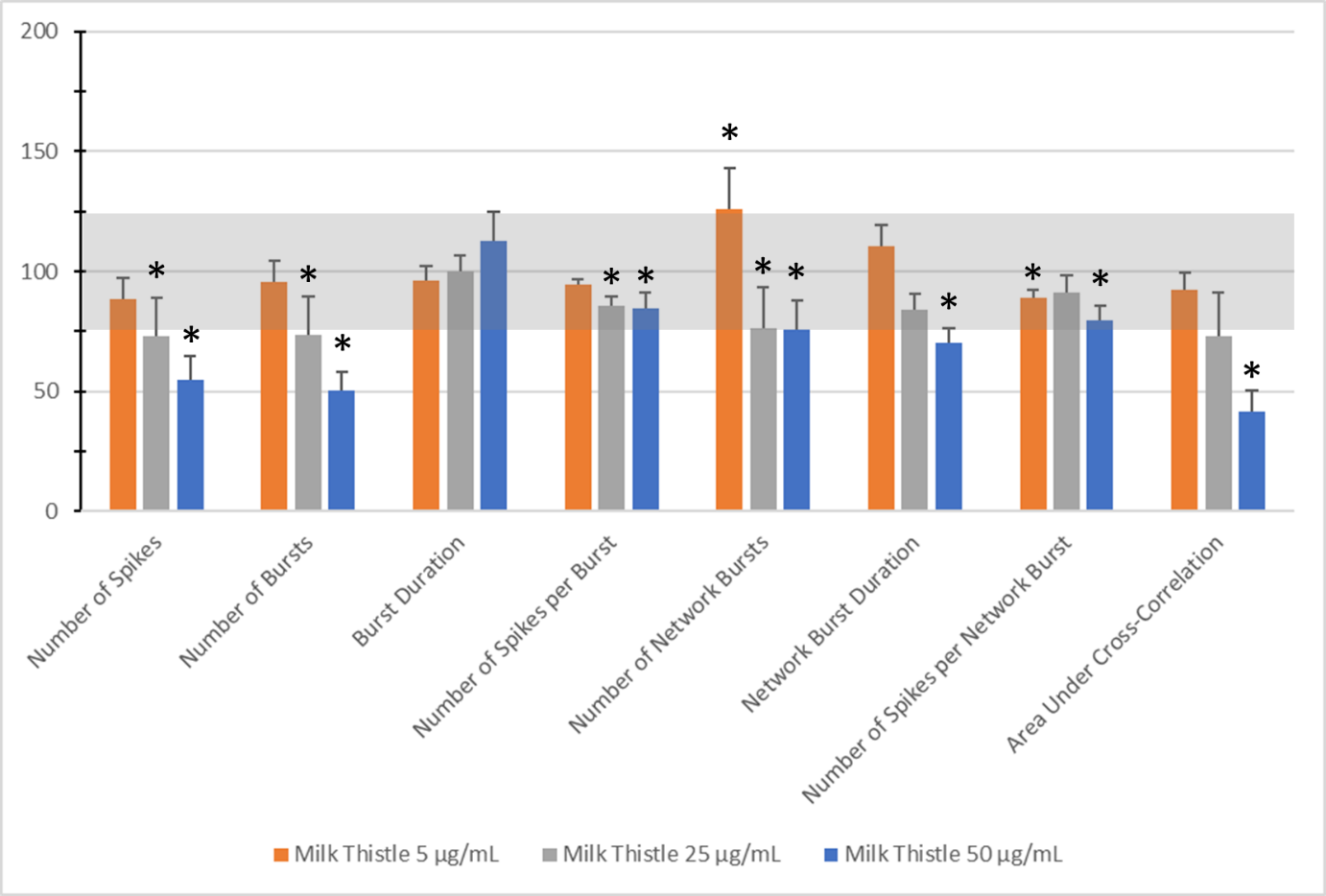


**Figure S1. Overview of the effects of exposure to Milk thistle extract on eight neuronal activity parameters.** Effects of acute exposure to the botanical extracts at doses from 50 µg/mL to 5 µg/mL on neuronal activity in rat primary cortical cultures grown on MEA are expressed as mean + SEM (from n=14-18 wells, N=2-3 plates) normalized to DMSO control. Values that do not exceed the BMR of 25%, indicated by the light grey area, are considered to be of limited toxicological relevance. Asterisks indicate values that deviate significantly from DMSO control (p<0.05).


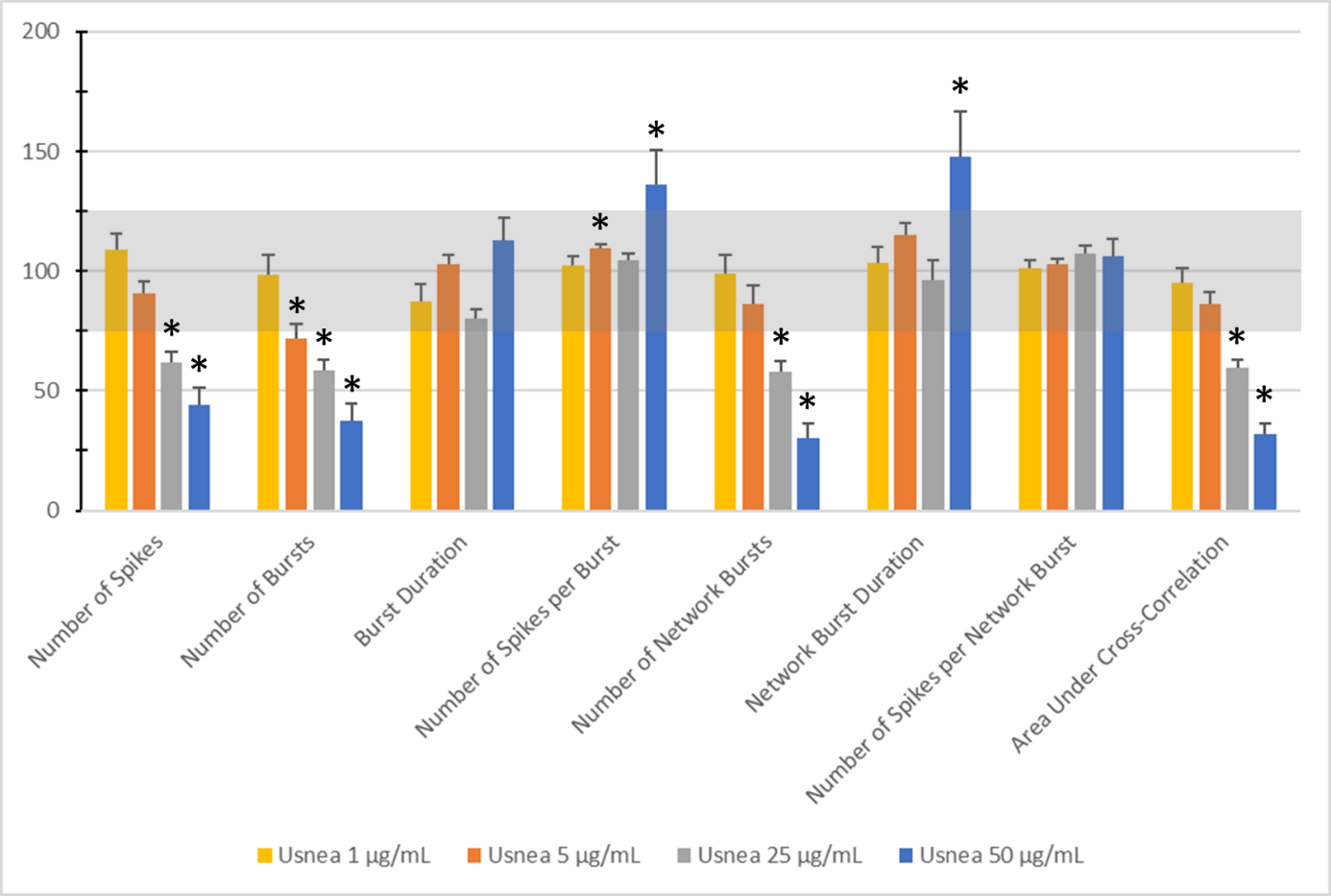


**Figure S2. Overview of the effects of exposure to usnea extract on eight neuronal activity parameters.** Effects of acute exposure to the botanical extracts at doses from 50 µg/mL to 5 µg/mL on neuronal activity in rat primary cortical cultures grown on MEA are expressed as mean + SEM (from n=16-26 wells, N=3-5 plates) normalized to DMSO control. Values that do not exceed the BMR of 25%, indicated by the light grey area, are considered to be of limited toxicological relevance. Asterisks indicate values that deviate significantly from DMSO control (p<0.05).


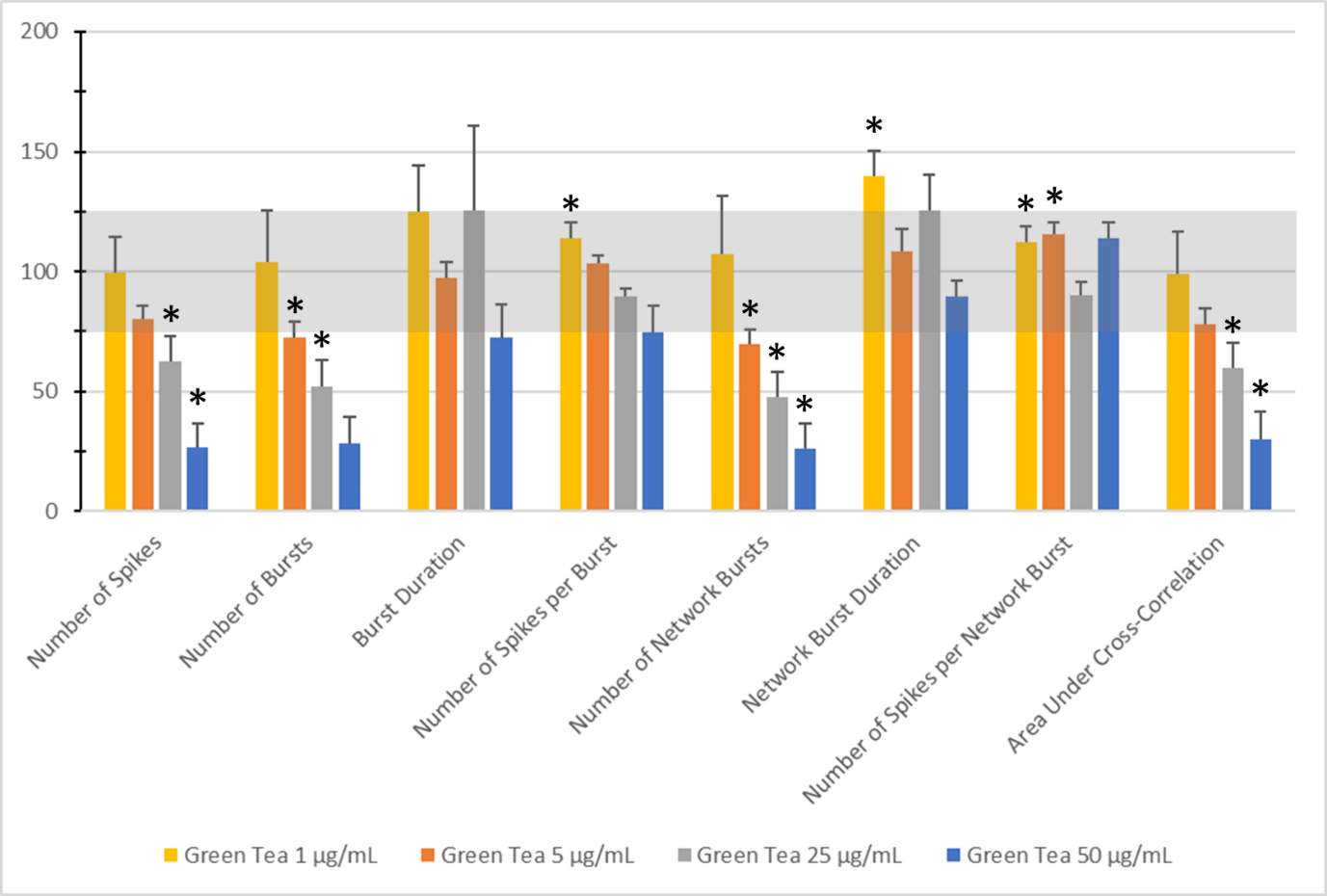


**Figure S3. Overview of the effects of exposure to green tea extract on eight neuronal activity parameters.** Effects of acute exposure to the botanical extracts at doses from 50 µg/mL to 1 µg/mL on neuronal activity in rat primary cortical cultures grown on MEA are expressed as mean + SEM (from n=11-17 wells, N=2-3 plates) normalized to DMSO control. Values that do not exceed the BMR of 25%, indicated by the light grey area, are considered to be of limited toxicological relevance. Asterisks indicate values that deviate significantly from DMSO control (p<0.05).


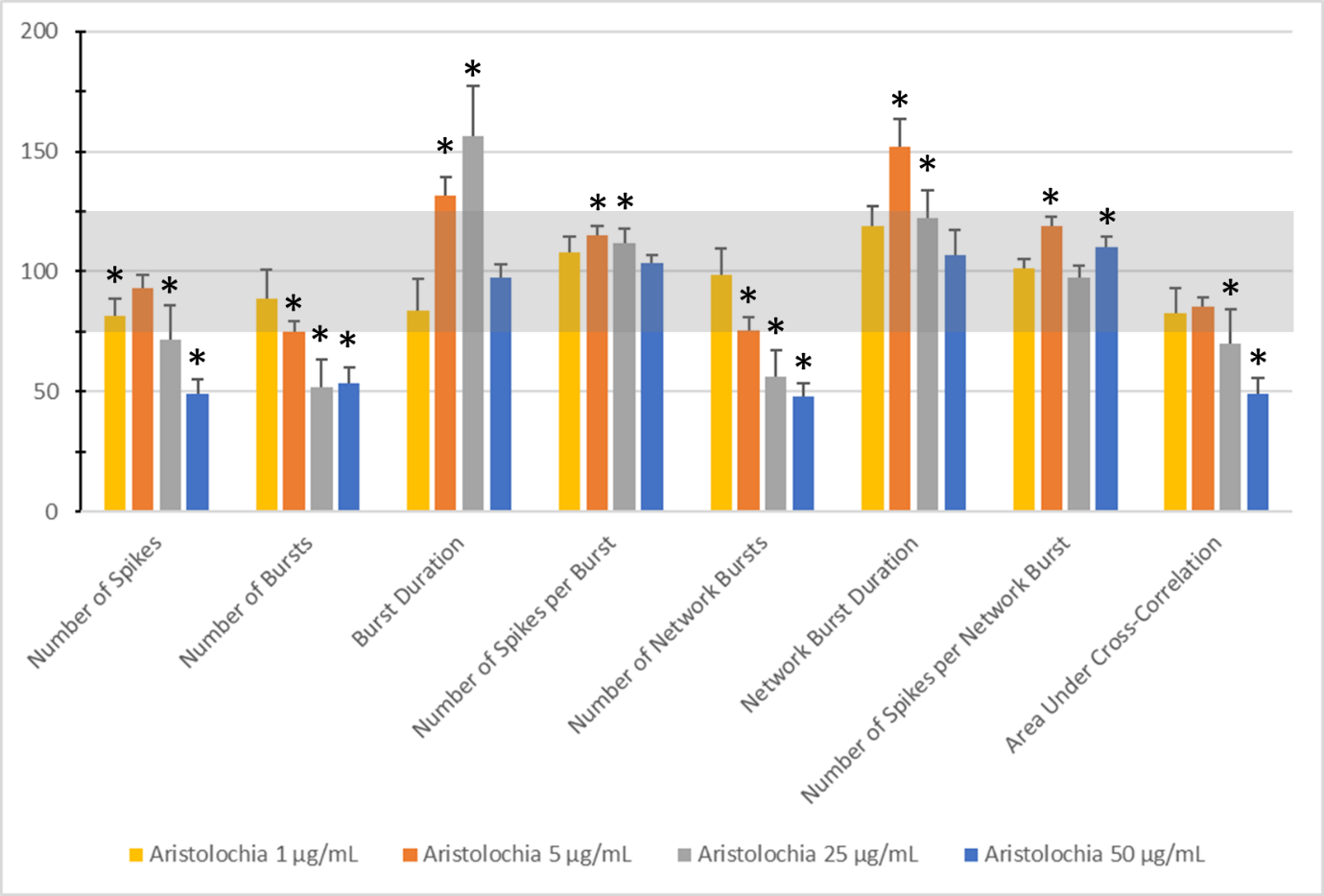


**Figure S4. Overview of the effects of exposure to aristolochia extract on eight neuronal activity parameters.** Effects of acute exposure to the botanical extracts at doses from 50 µg/mL to 1 µg/mL on neuronal activity in rat primary cortical cultures grown on MEA are expressed as mean + SEM (from n=15-22 wells, N=2-3 plates) normalized to DMSO control. Values that do not exceed the BMR of 25%, indicated by the light grey area, are considered to be of limited toxicological relevance. Asterisks indicate values that deviate significantly from DMSO control (p<0.05).


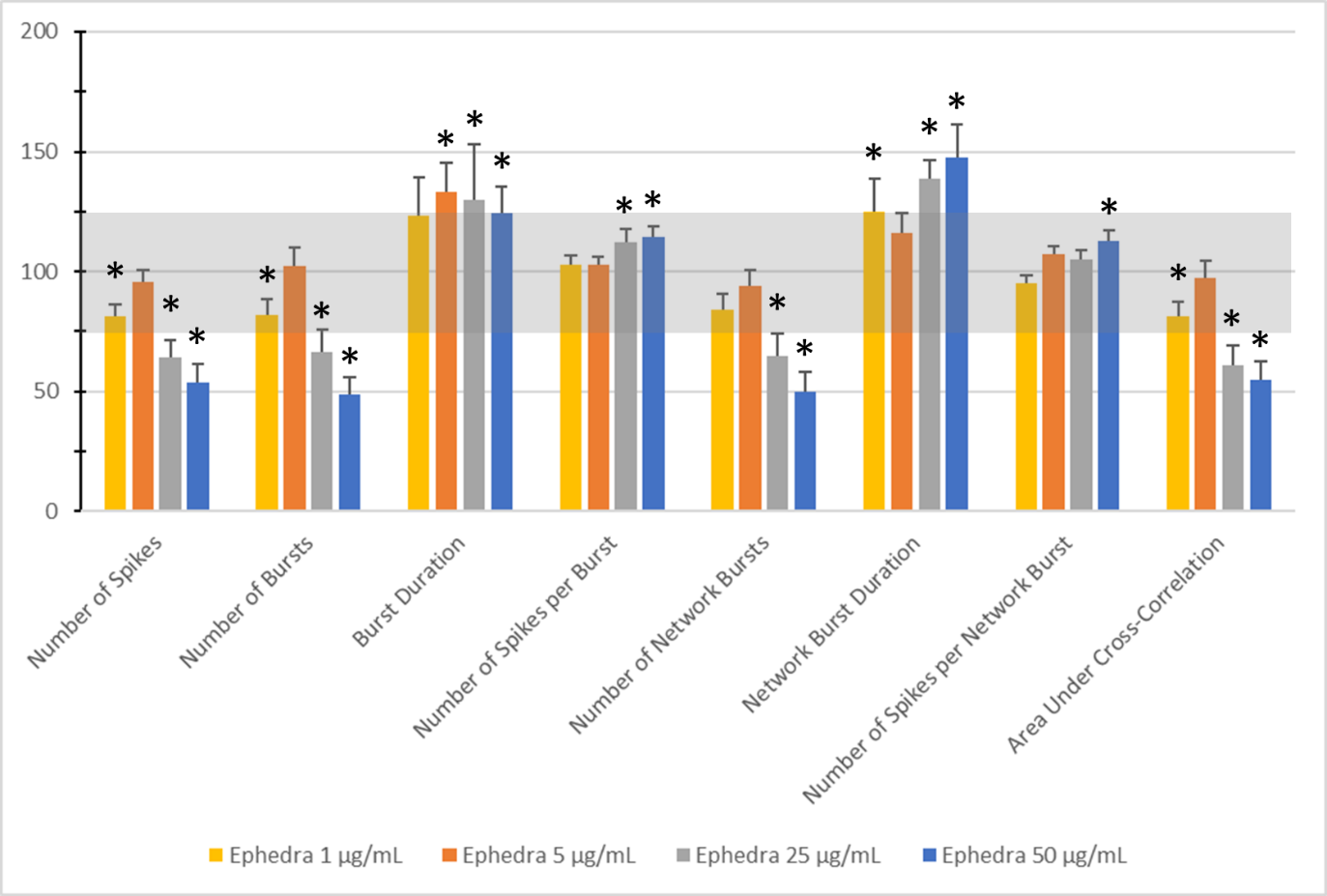


**Figure S5. Overview of the effects of exposure to ephedra extract on eight neuronal activity parameters.** Effects of acute exposure to the botanical extracts at doses from 50 µg/mL to 1 µg/mL on neuronal activity in rat primary cortical cultures grown on MEA are expressed as mean + SEM (from n=28-41 wells, N=6 plates) normalized to DMSO control. Values that do not exceed the BMR of 25%, indicated by the light grey area, are considered to be of limited toxicological relevance. Asterisks indicate values that deviate significantly from DMSO control (p<0.05).


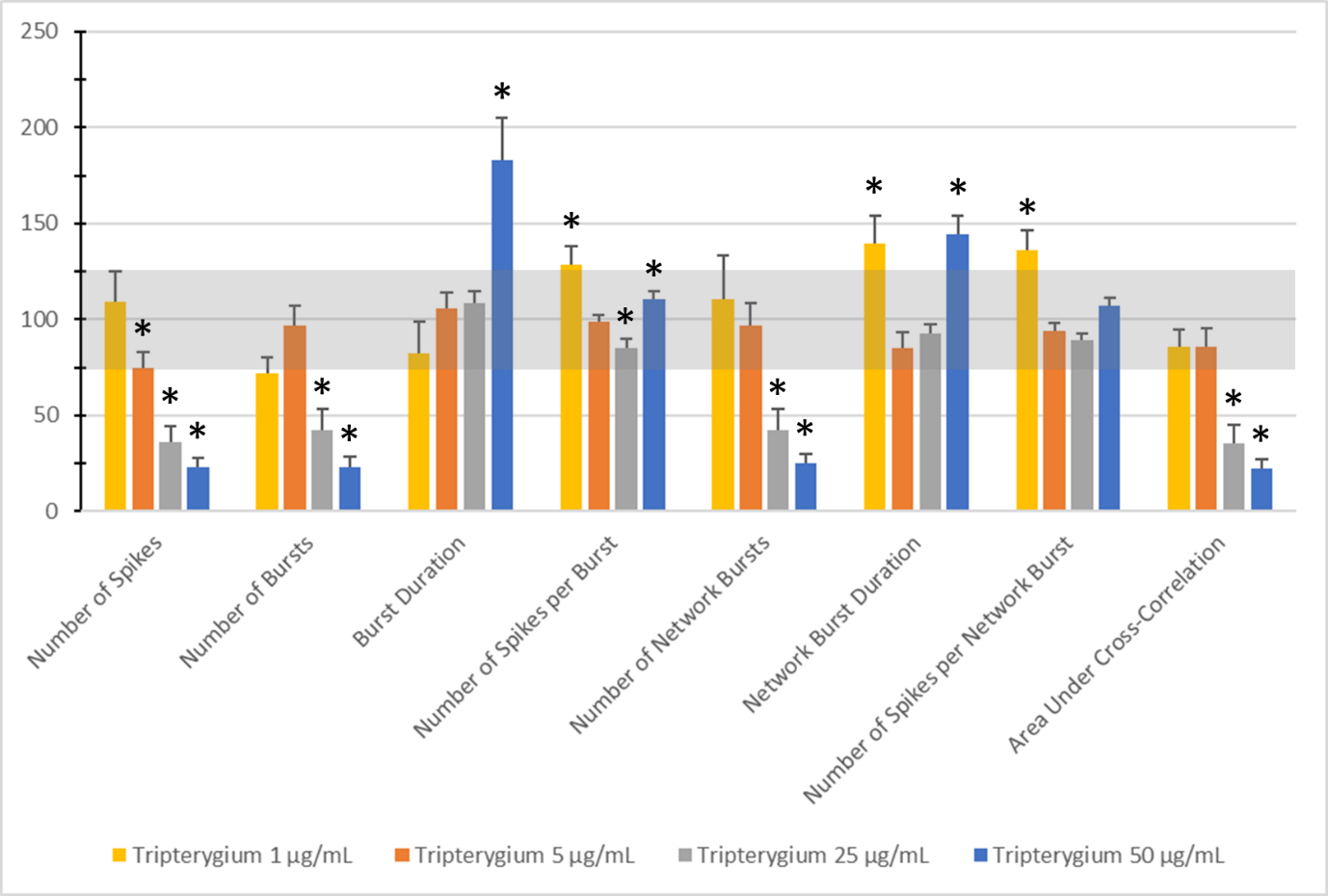


**Figure S6. Overview of the effects of exposure to tripterygium extract on eight neuronal activity parameters.** Effects of acute exposure to the botanical extracts at doses from 50 µg/mL to 1 µg/mL on neuronal activity in rat primary cortical cultures grown on MEA are expressed as mean + SEM (from n=11-25 wells, N=2-3 plates) normalized to DMSO control. Values that do not exceed the BMR of 25%, indicated by the light grey area, are considered to be of limited toxicological relevance. Asterisks indicate values that deviate significantly from DMSO control (p<0.05).


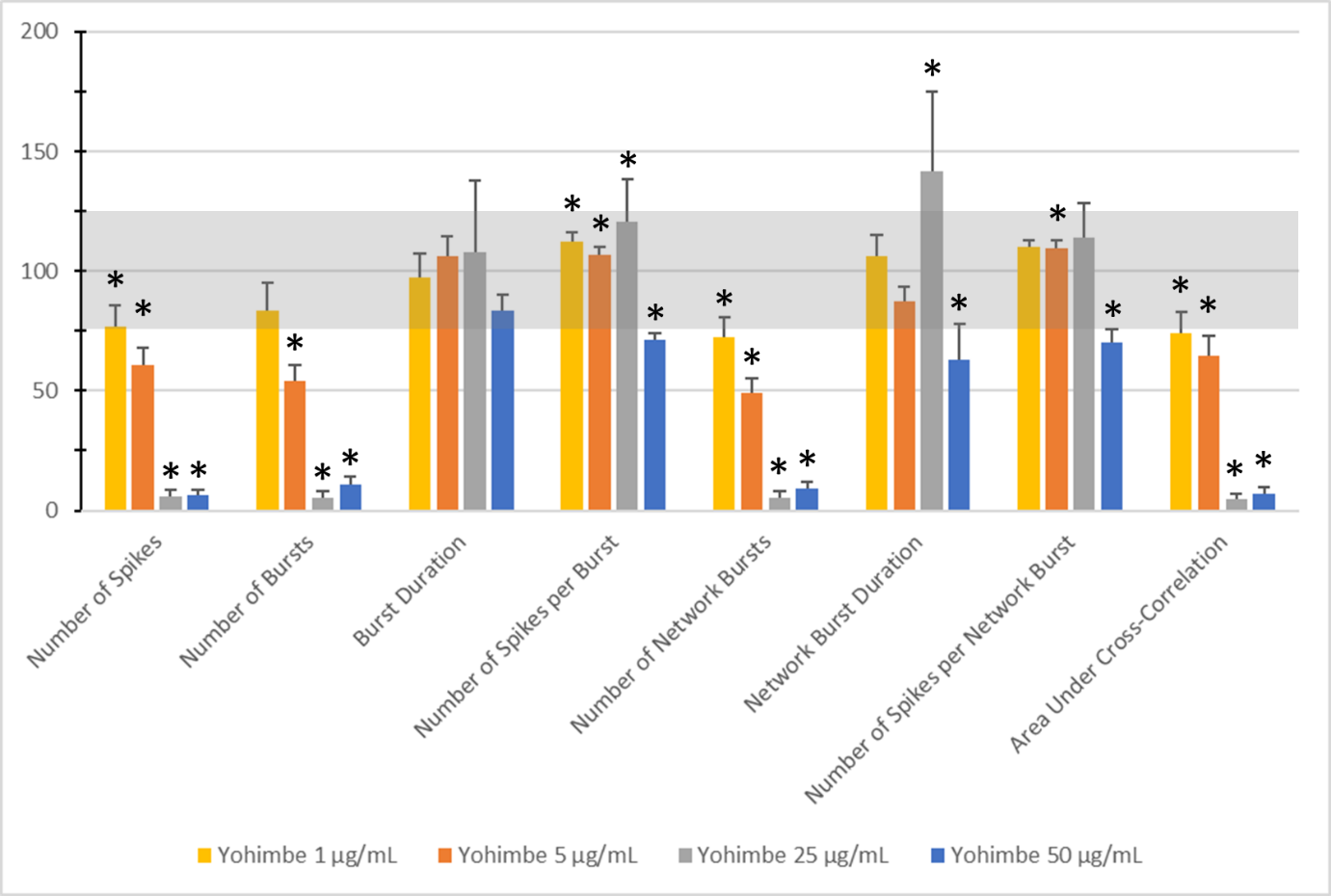


**Figure S7. Overview of the effects of exposure to yohimbe extract on eight neuronal activity parameters.** Effects of acute exposure to the botanical extracts at doses from 50 µg/mL to 1 µg/mL on neuronal activity in rat primary cortical cultures grown on MEA are expressed as mean + SEM (from n=14-36 wells, N=2-5 plates) normalized to DMSO control. Values that do not exceed the BMR of 25%, indicated by the light grey area, are considered to be of limited toxicological relevance. Asterisks indicate values that deviate significantly from DMSO control (p<0.05).


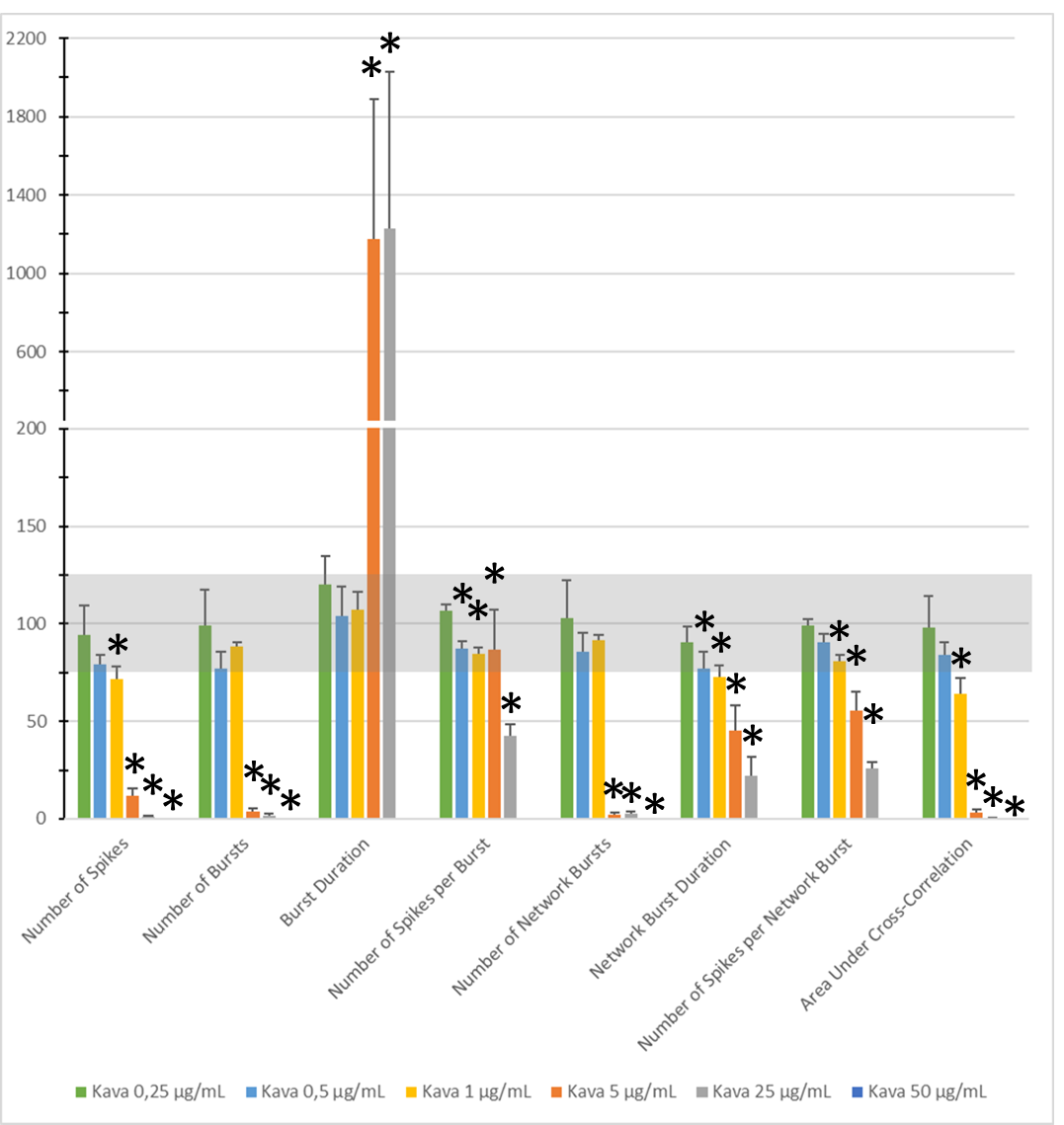


**Figure S8. Overview of the effects of exposure to Kava extract on eight neuronal activity parameters.** Effects of acute exposure to the botanical extracts at doses from 50 µg/mL to 0,25 µg/mL on neuronal activity in rat primary cortical cultures grown on MEA are expressed as mean + SEM (from n=12-18 wells, N=2 plates) normalized to DMSO control. Values that do not exceed the BMR of 25%, indicated by the light grey area, are considered to be of limited toxicological relevance. Asterisks indicate values that deviate significantly from DMSO control (p<0.05).


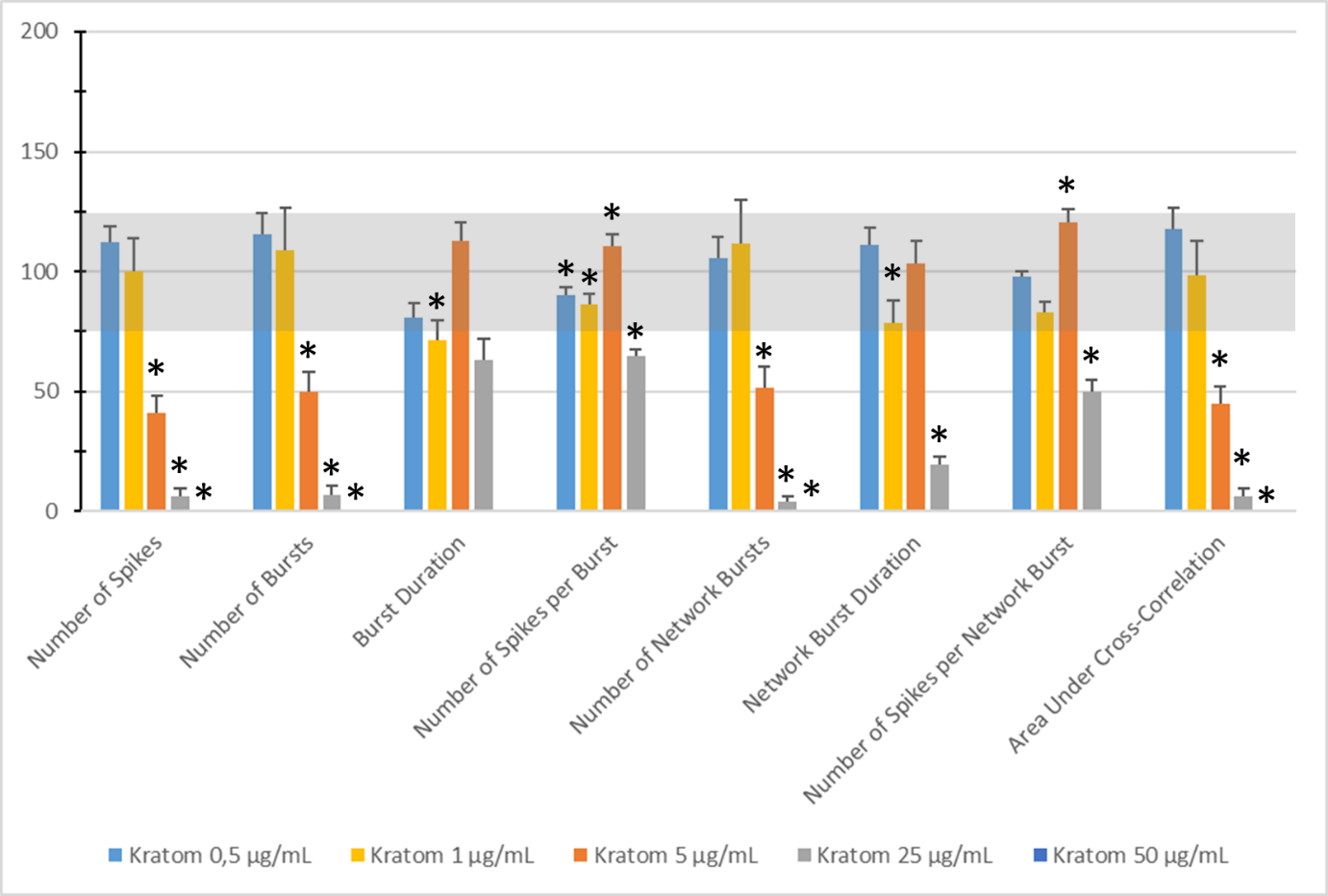


**Figure S9. Overview of the effects of exposure to kratom extract on eight neuronal activity parameters.** Effects of acute exposure to the botanical extracts at doses from 50 µg/mL to 0,5 µg/mL on neuronal activity in rat primary cortical cultures grown on MEA are expressed as mean + SEM (from n=13-23 wells, N=2-4 plates) normalized to DMSO control. Values that do not exceed the BMR of 25%, indicated by the light grey area, are considered to be of limited toxicological relevance. Asterisks indicate values that deviate significantly from DMSO control (p<0.05).

**
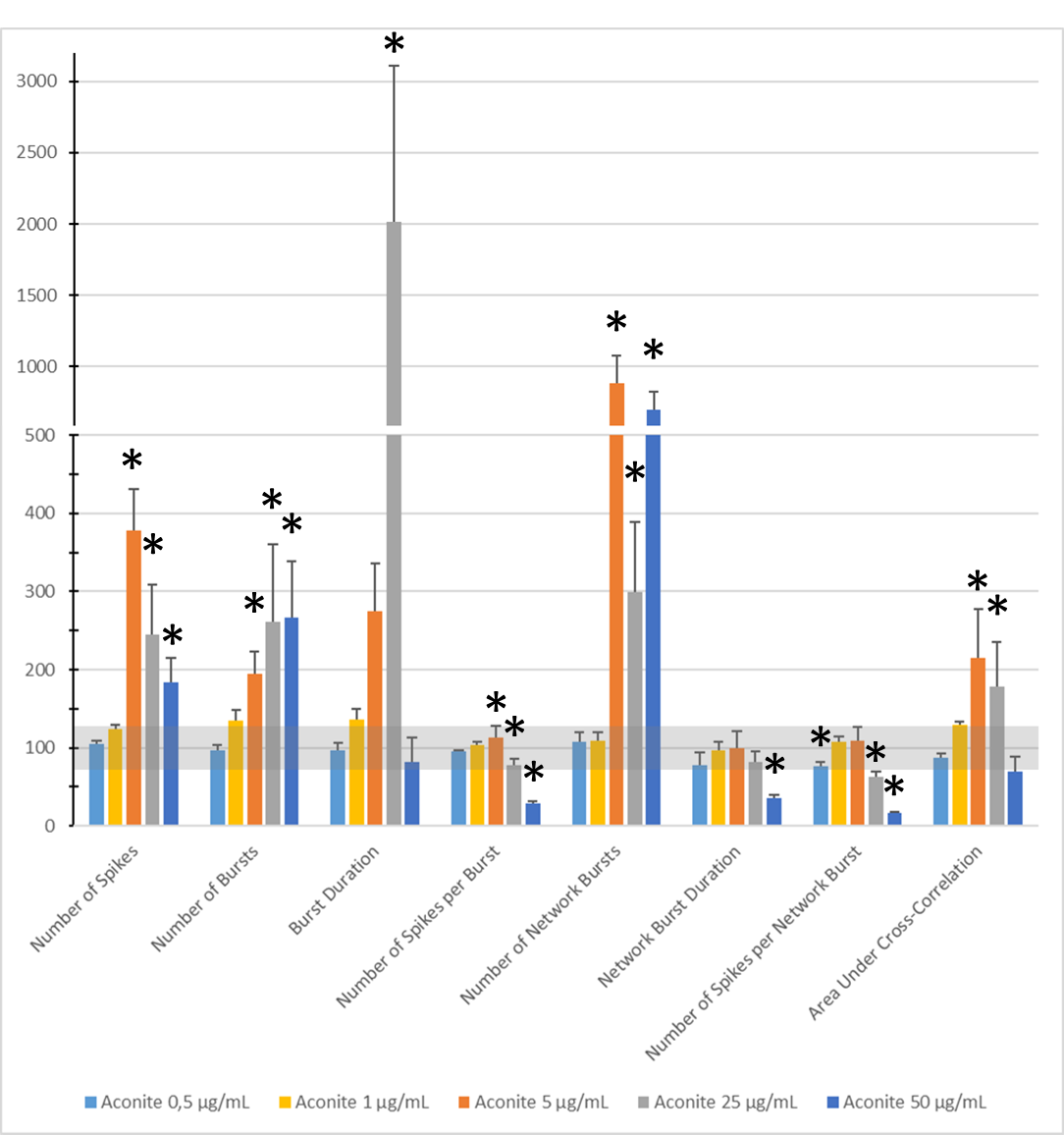
**

**Figure S10. Overview of the effects of exposure to aconite extract on eight neuronal activity parameters.** Effects of acute exposure to the botanical extracts at doses from 50 µg/mL to 0,5 µg/mL on neuronal activity in rat primary cortical cultures grown on MEA are expressed as mean + SEM (from n=12-20 wells, N=2-3 plates) normalized to DMSO control. Values that do not exceed the BMR of 25%, indicated by the light grey area, are considered to be of limited toxicological relevance. Asterisks indicate values that deviate significantly from DMSO control (p<0.05).


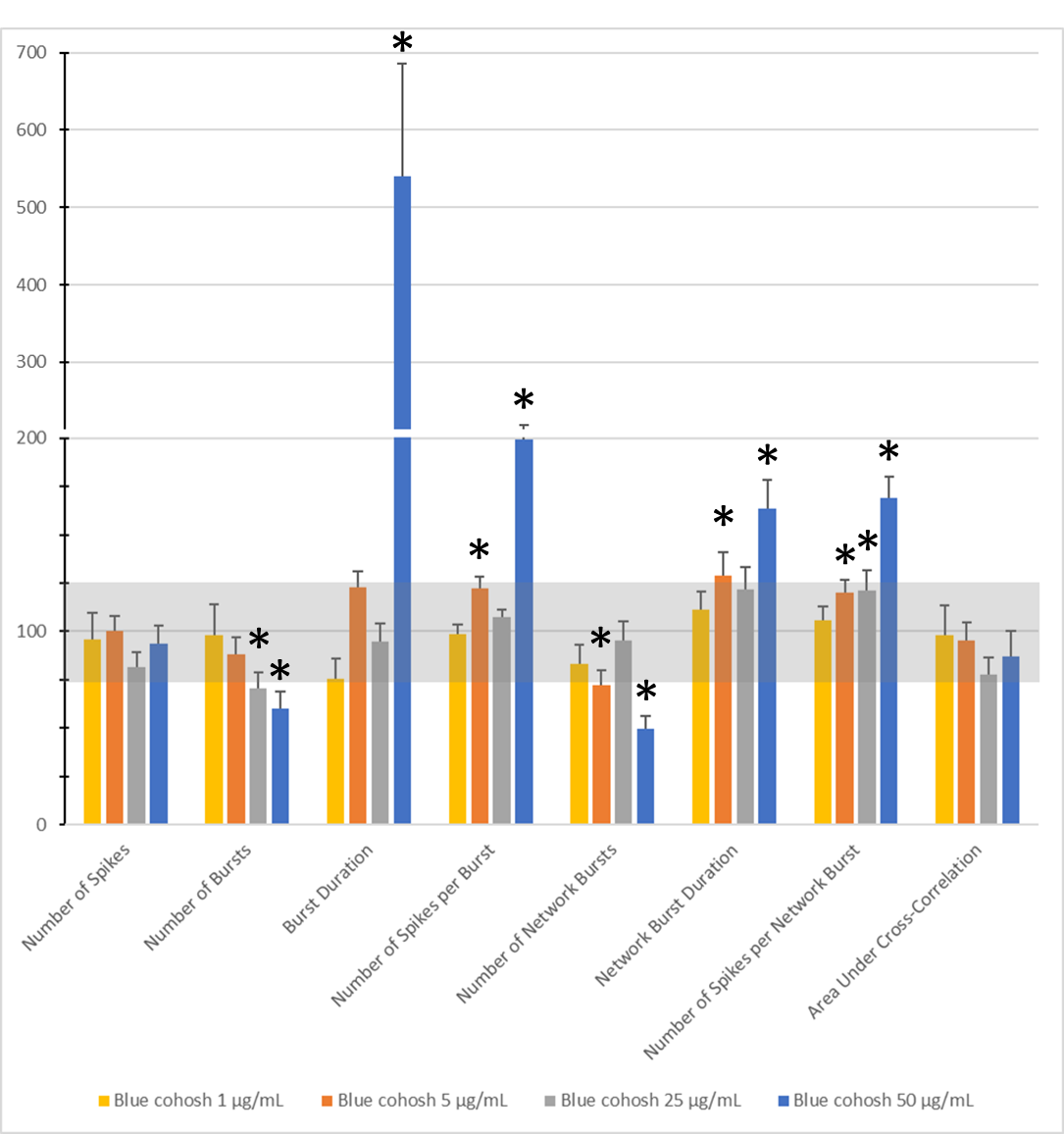


**Figure S11. Overview of the effects of exposure to blue cohosh extract on eight neuronal activity parameters.** Effects of acute exposure to the botanical extracts at doses from 50 µg/mL to 1 µg/mL on neuronal activity in rat primary cortical cultures grown on MEA are expressed as mean + SEM (from n=14-20 wells, N=2-3 plates) normalized to DMSO control. Values that do not exceed the BMR of 25%, indicated by the light grey area, are considered to be of limited toxicological relevance. Asterisks indicate values that deviate significantly from DMSO control (p<0.05).


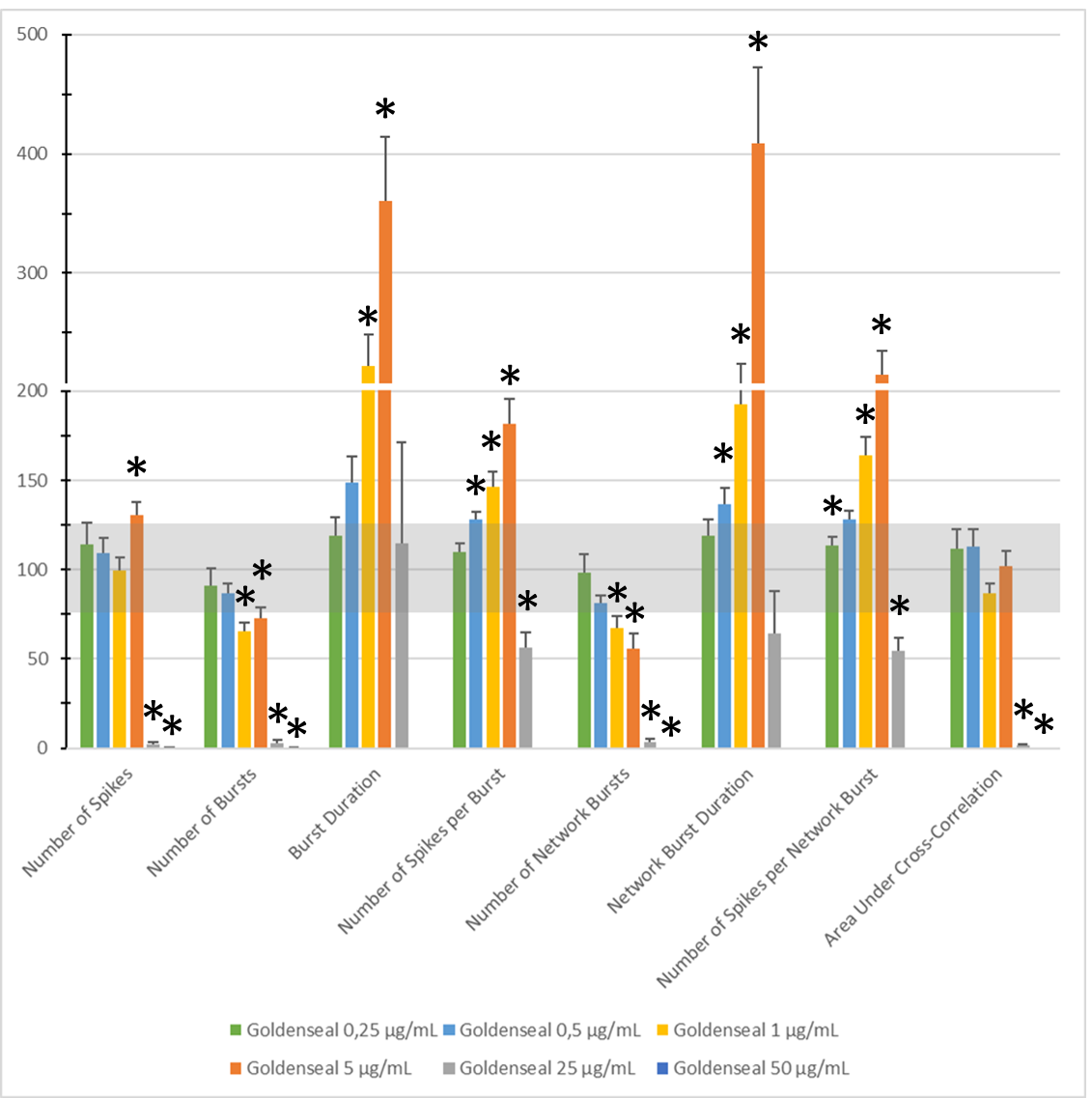


**Figure S12. Overview of the effects of exposure to goldenseal extract on eight neuronal activity parameters.** Effects of acute exposure to the botanical extracts at doses from 50 µg/mL to 0,25 µg/mL on neuronal activity in rat primary cortical cultures grown on MEA are expressed as mean + SEM (from n=11-15 wells, N=2 plates) normalized to DMSO control. Values that do not exceed the BMR of 25%, indicated by the light grey area, are considered to be of limited toxicological relevance. Asterisks indicate values that deviate significantly from DMSO control (p<0.05).


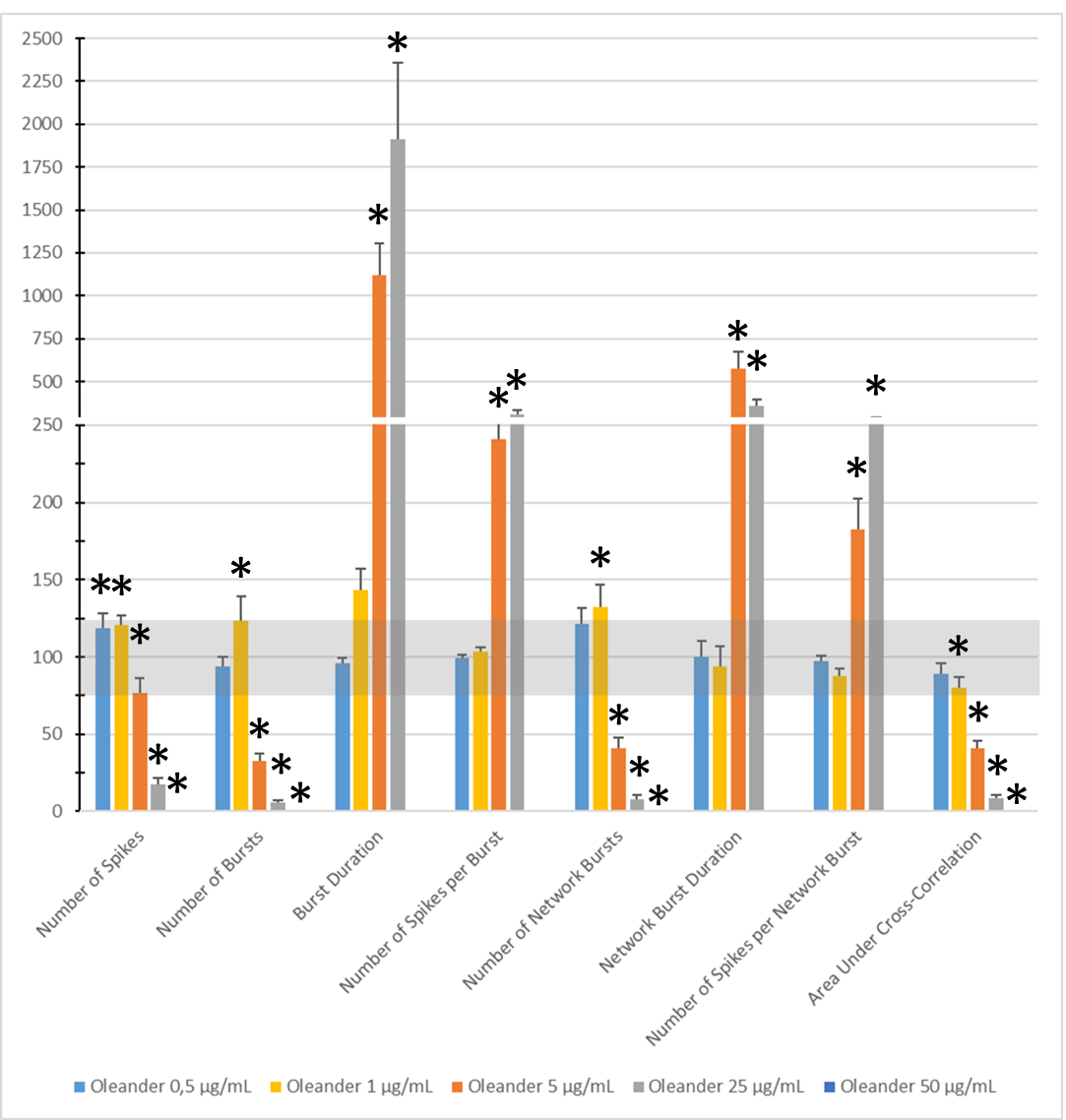


**Figure S13. Overview of the effects of exposure to oleander extract on eight neuronal activity parameters.** Effects of acute exposure to the botanical extracts at doses from 50 µg/mL to 0,5 µg/mL on neuronal activity in rat primary cortical cultures grown on MEA are expressed as mean + SEM (from n=9-34 wells, N=2-5 plates) normalized to DMSO control. Values that do not exceed the BMR of 25%, indicated by the light grey area, are considered to be of limited toxicological relevance. Asterisks indicate values that deviate significantly from DMSO control (p<0.05).


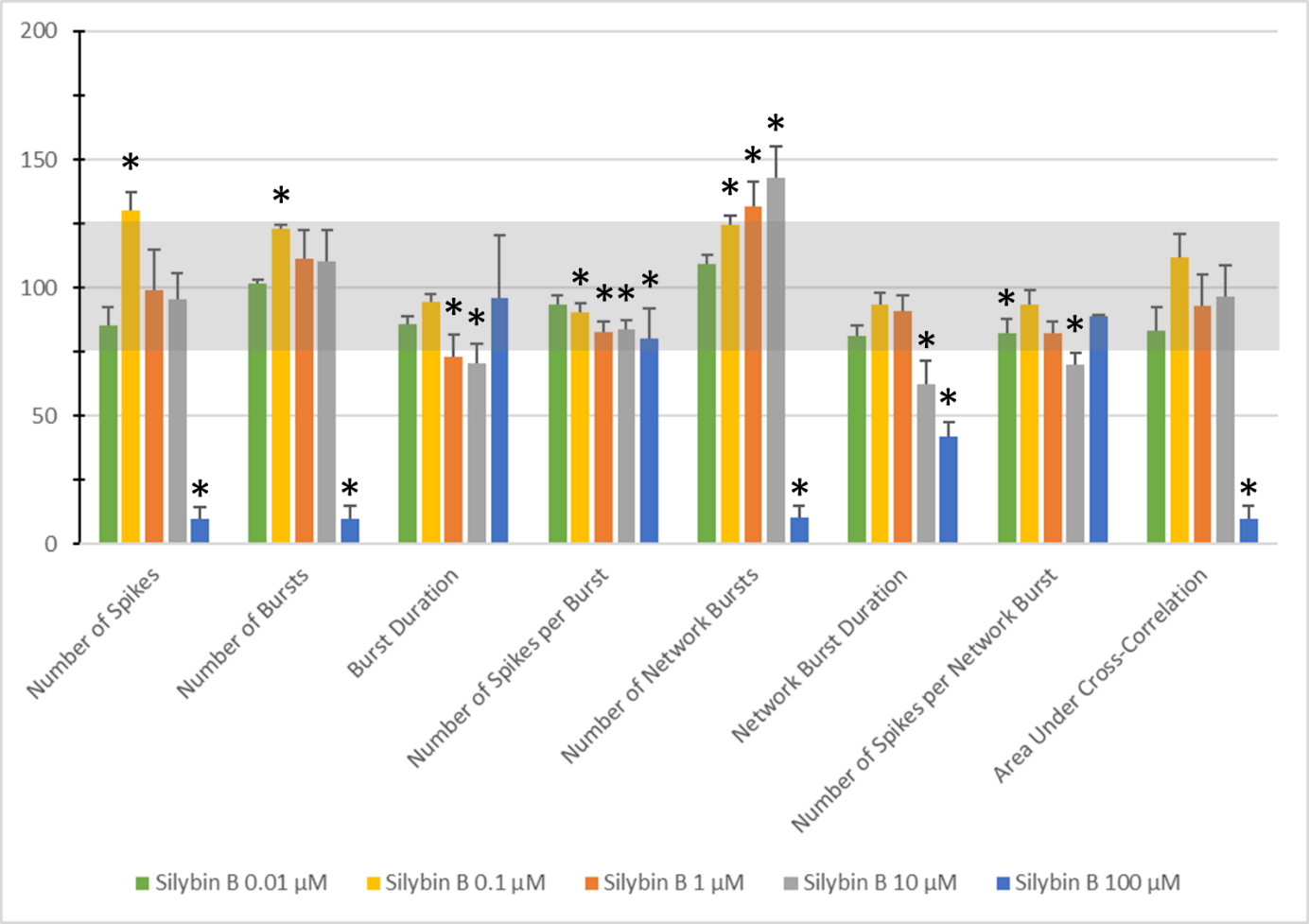


**Figure S14. Overview of the effects of exposure to silybin B on eight neuronal activity parameters.** Effects of acute exposure to silybin B at concentrations from 0.1 - 100 µM on neuronal activity in rat primary cortical cultures grown on MEA are expressed as mean + SEM (from n=13-43 wells, N=2-6 plates) normalized to DMSO control. Values that do not exceed the BMR of 25%, indicated by the light grey area, are considered to be of limited toxicological relevance. Asterisks indicate values that deviate significantly from DMSO control (p<0.05).


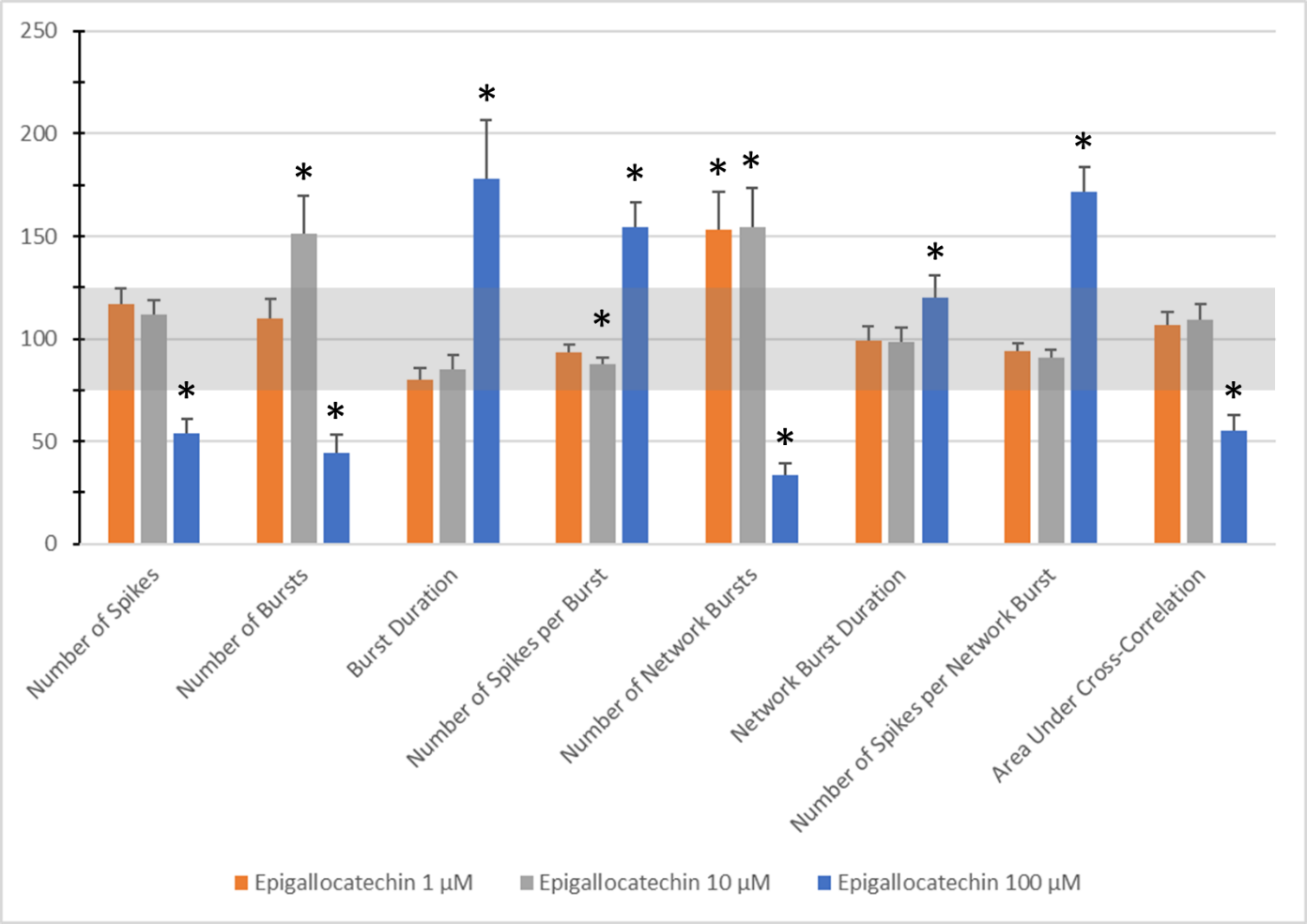


**Figure S15. Overview of the effects of exposure to epigallocatechin on eight neuronal activity parameters.** Effects of acute exposure to epigallocatechin at concentrations from 0.001 - 100 µM on neuronal activity in rat primary cortical cultures grown on MEA are expressed as mean + SEM (from n=20-41 wells, N=3-6 plates) normalized to DMSO control. Values that do not exceed the BMR of 25%, indicated by the light grey area, are considered to be of limited toxicological relevance. Asterisks indicate values that deviate significantly from DMSO control (p<0.05).


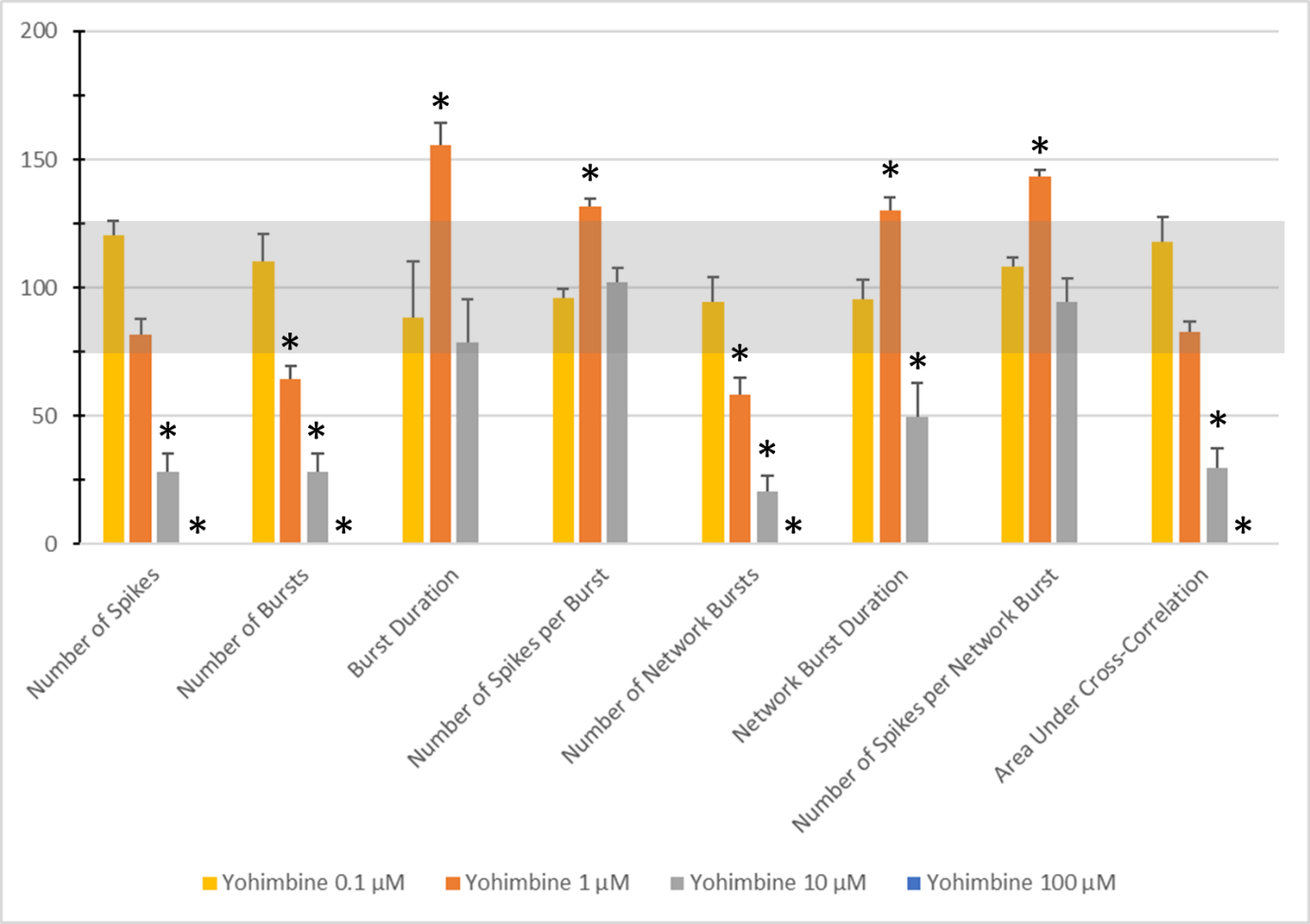


**Figure S16. Overview of the effects of exposure to yohimbine on eight neuronal activity parameters.** Effects of acute exposure to yohimbine at concentrations from 0.1 - 100 µM on neuronal activity in rat primary cortical cultures grown on MEA are expressed as mean + SEM (from n=7-23 wells, N=1-3 plates) normalized to DMSO control. Values that do not exceed the BMR of 25%, indicated by the light grey area, are considered to be of limited toxicological relevance. Asterisks indicate values that deviate significantly from DMSO control (p<0.05).


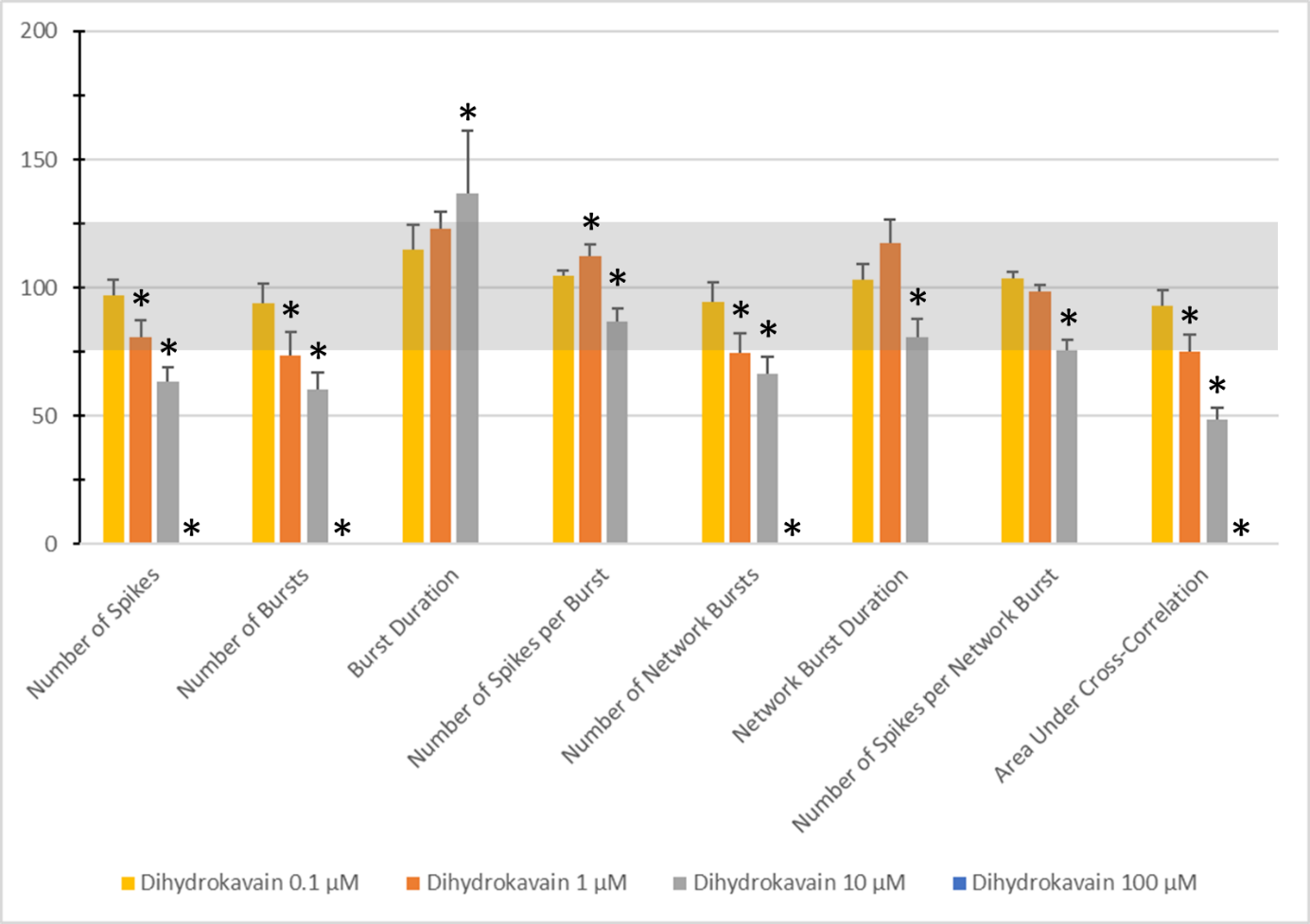


**Figure S17. Overview of the effects of exposure to dihydrokavain on eight neuronal activity parameters.** Effects of acute exposure to dihydrokavain at concentrations from 0.0001 - 100 µM on neuronal activity in rat primary cortical cultures grown on MEA are expressed as mean + SEM (from n=13-29 wells, N=2-4 plates) normalized to DMSO control. Values that do not exceed the BMR of 25%, indicated by the light grey area, are considered to be of limited toxicological relevance. Asterisks indicate values that deviate significantly from DMSO control (p<0.05).


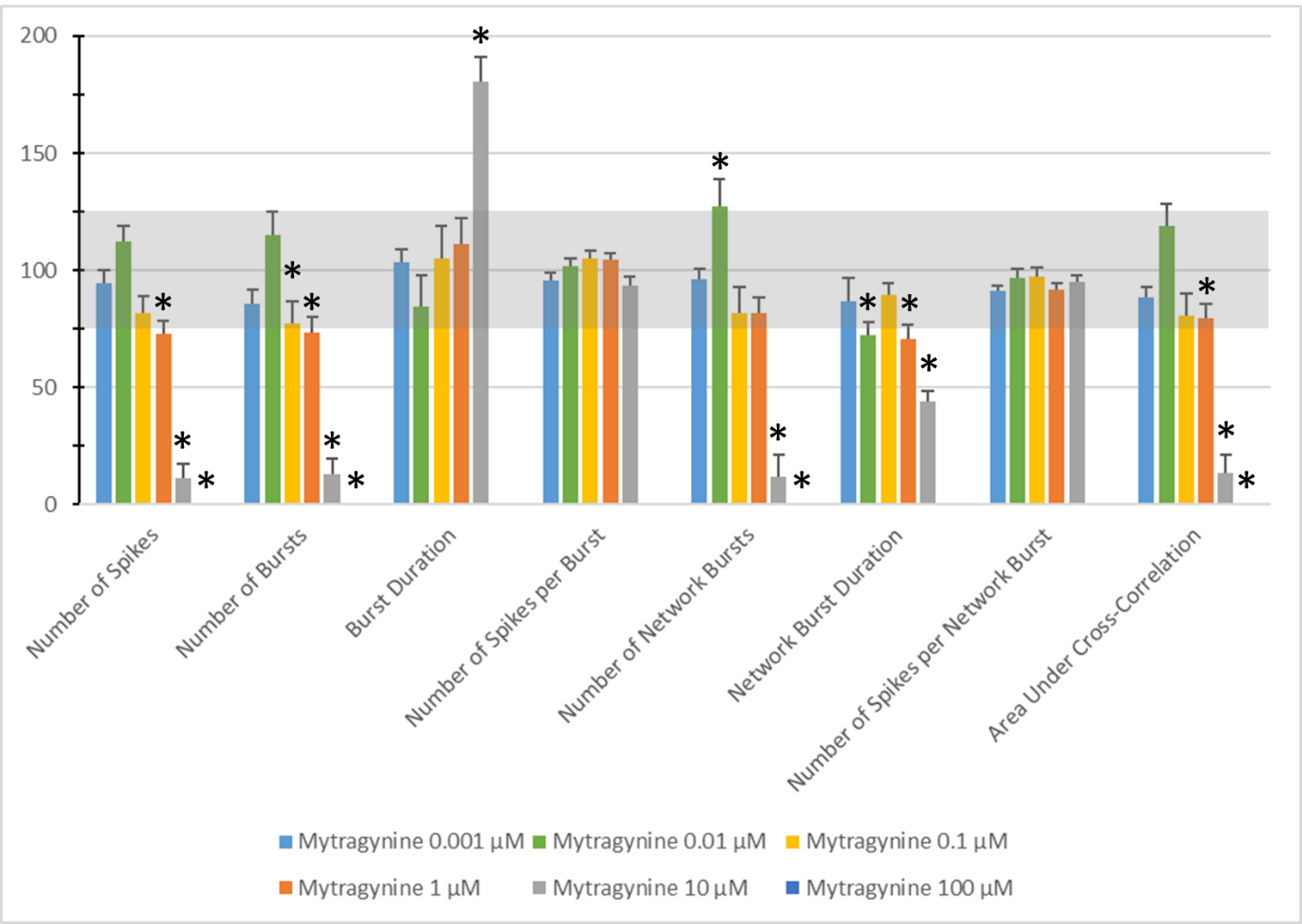


**Figure S18. Overview of the effects of exposure to mytragynine on eight neuronal activity parameters.** Effects of acute exposure to mytragynine at concentrations from 0.001 - 100 µM on neuronal activity in rat primary cortical cultures grown on MEA are expressed as mean + SEM (from n=7-30 wells, N=1-4 plates) normalized to DMSO control. Values that do not exceed the BMR of 25%, indicated by the light grey area, are considered to be of limited toxicological relevance. Asterisks indicate values that deviate significantly from DMSO control (p<0.05).


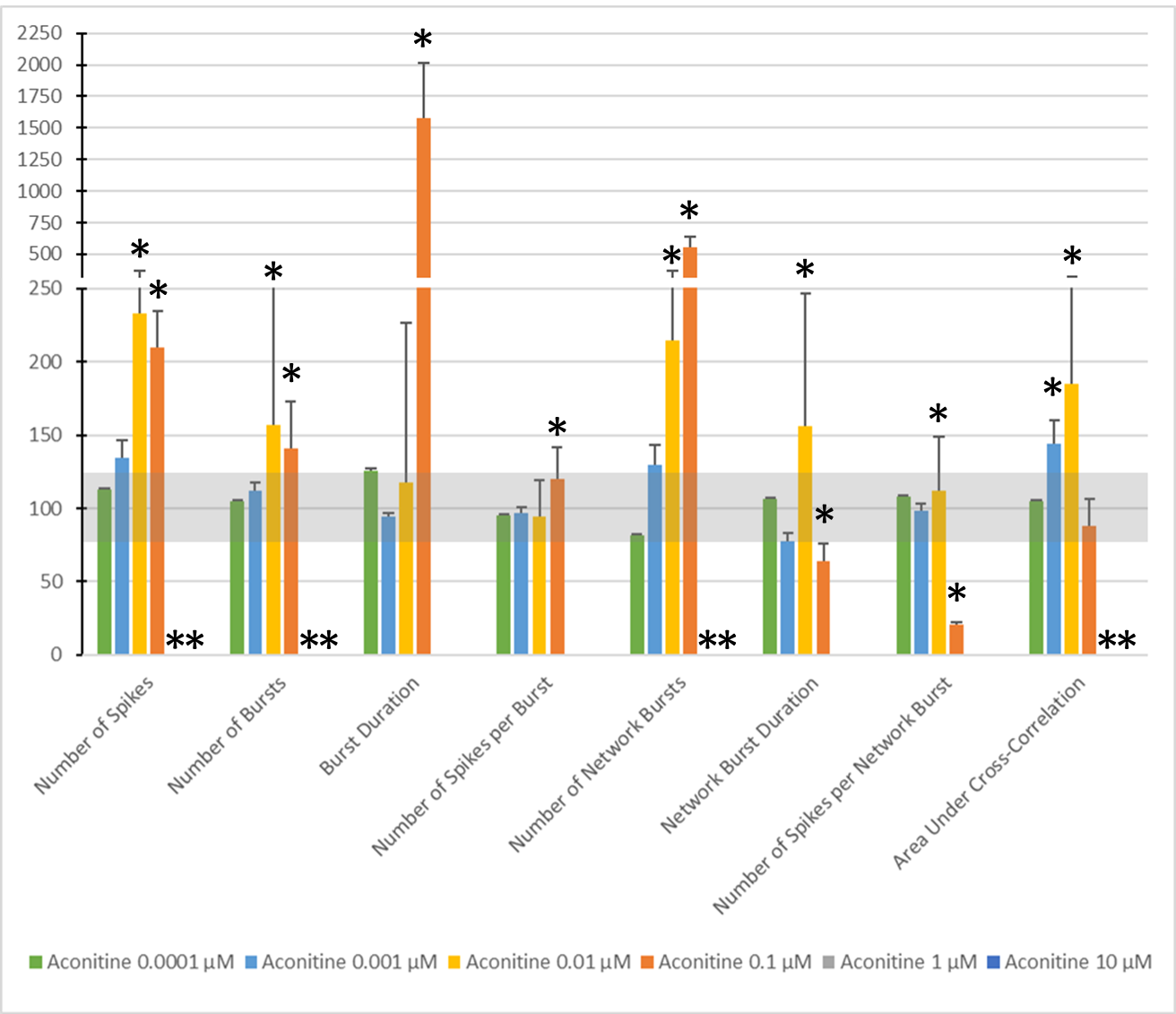


**Figure S19. Overview of the effects of exposure to aconitine on eight neuronal activity parameters.** Effects of acute exposure to aconitine at concentrations from 0.0001 - 10 µM on neuronal activity in rat primary cortical cultures grown on MEA are expressed as mean + SEM (from n=15-42 wells, N=2-6 plates) normalized to DMSO control. Values that do not exceed the BMR of 25%, indicated by the light grey area, are considered to be of limited toxicological relevance. Asterisks indicate values that deviate significantly from DMSO control (p<0.05).


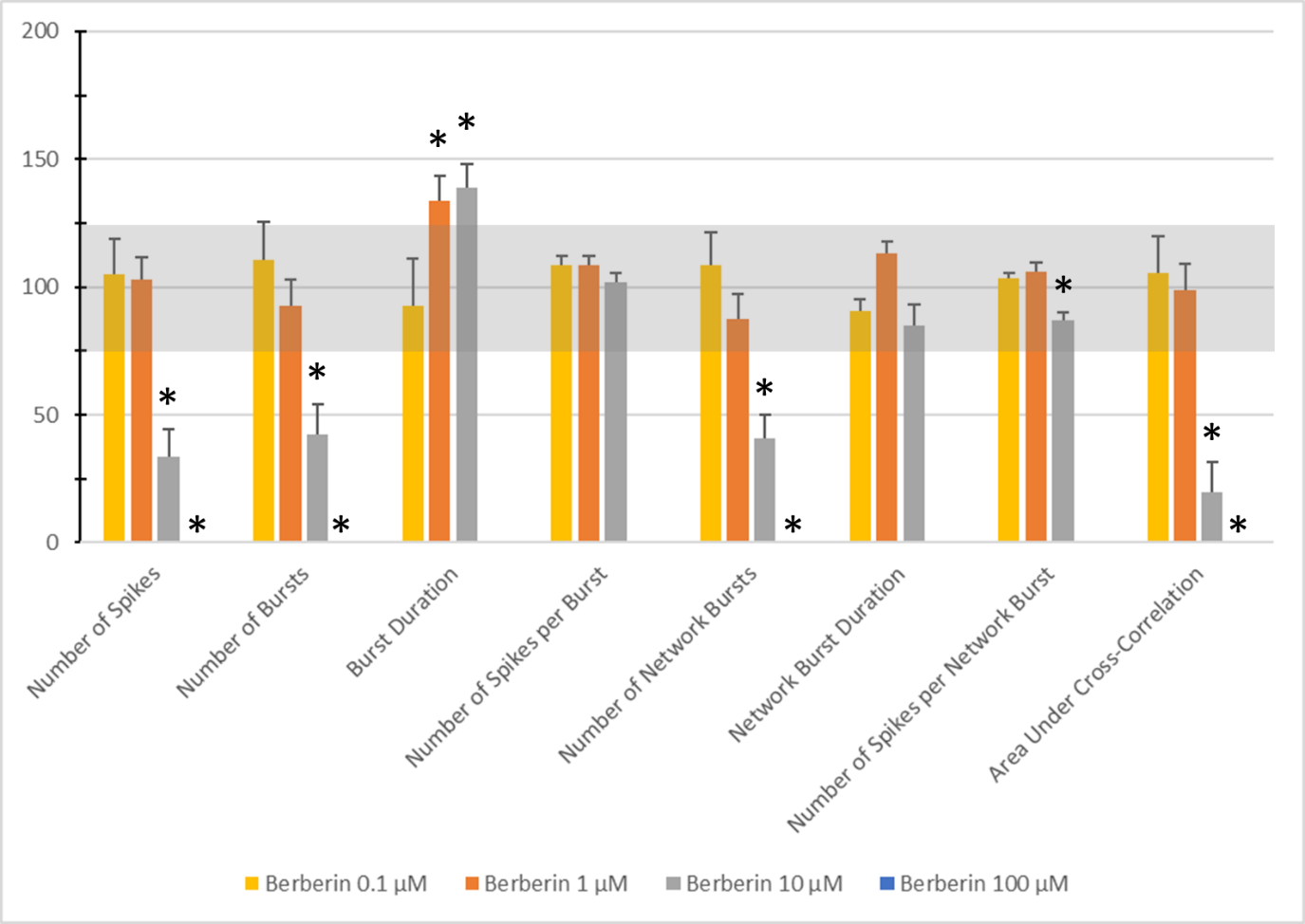


**Figure S20. Overview of the effects of exposure to berberine on eight neuronal activity parameters.** Effects of acute exposure to berberine at concentrations from 0.1 - 100 µM on neuronal activity in rat primary cortical cultures grown on MEA are expressed as mean + SEM (from n=6-39 wells, N=1-5 plates) normalized to DMSO control. Values that do not exceed the BMR of 25%, indicated by the light grey area, are considered to be of limited toxicological relevance. Asterisks indicate values that deviate significantly from DMSO control (p<0.05).


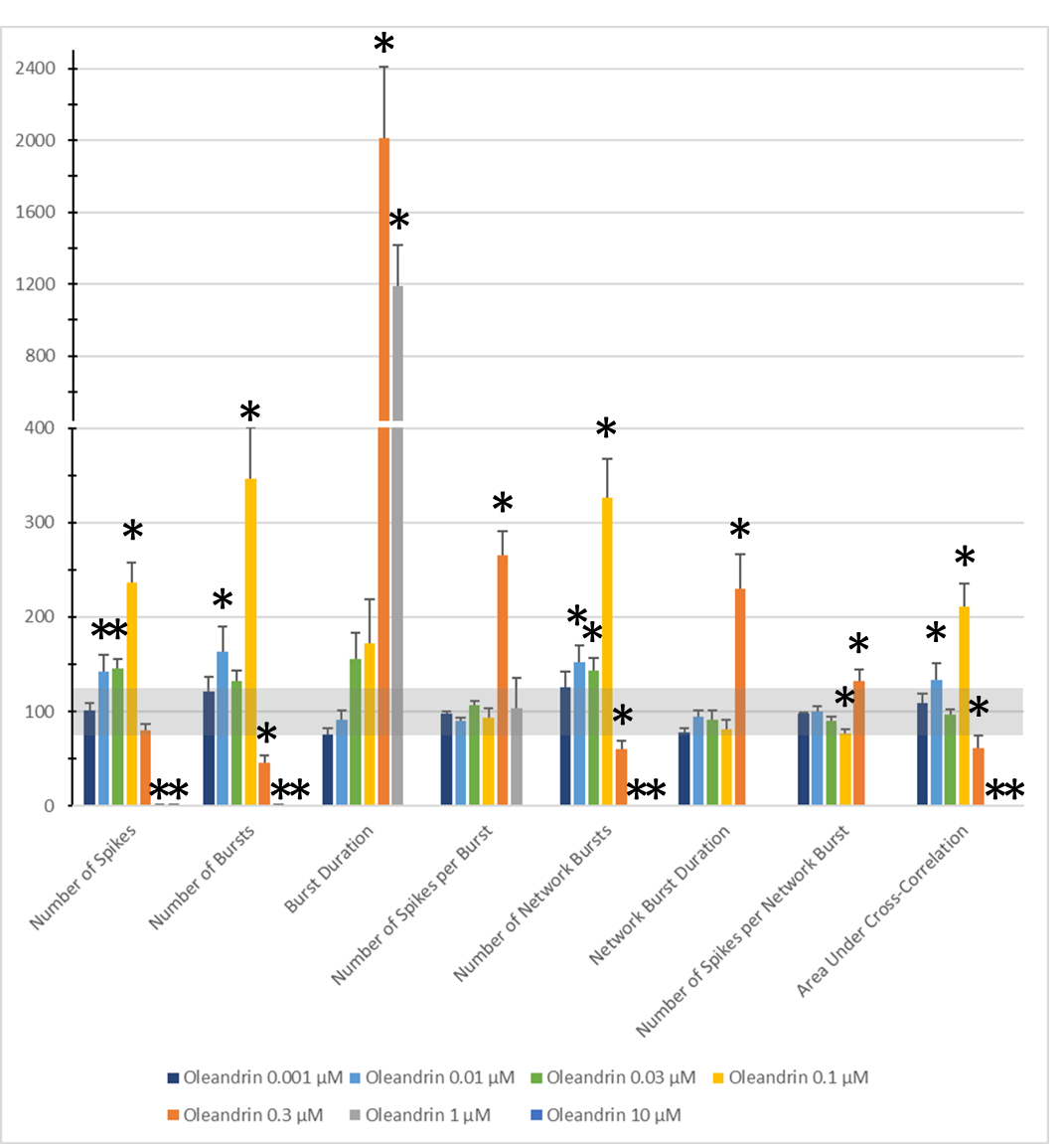


**Figure S21. Overview of the effects of exposure to oleandrin on eight neuronal activity parameters.** Effects of acute exposure to oleandrin at concentrations from 0.001 - 100 µM on neuronal activity in rat primary cortical cultures grown on MEA are expressed as mean + SEM (from n=7-34 wells, N=1-5 plates) normalized to DMSO control. Values that do not exceed the BMR of 25%, indicated by the light grey area, are considered to be of limited toxicological relevance. Asterisks indicate values that deviate significantly from DMSO control (p<0.05).

1. 1 Also commonly known as thunder god vine. [↑](#footnote-ref-1)
