# Supplementary material for: Acute uncomplicated urinary tract infections and subsequent type 2 diabetes diagnosis in women: a national cohort study including primary healthcare data
Source: Scand J Prim Health Care. 2025 Nov 6;44(1):1–5. doi: 10.1080/02813432.2025.2580905 (PMC12918306; doi:10.1080/02813432.2025.2580905)
Supplement: Supplemental Material [file IPRI_A_2580905_SM7501.pdf]

Chemical Analysis using LC-QToF

Plant Details:

NCNPR #	Accepted name	Family	Part	Collected by	TAXID (UoM)	Locality
2349	*Tripterygium wilfordii* Hook. f.	Celastraceae	Root	Trish	TAX0070010	Dongyang District Zhejiang Provience, China

Tripterygium wilfordii Hook. f.

- *Tripterygium wilfordii* Hook. F. (TWHF), commonly known as ‘Thunder God Vine’ (‘Lei-gong-teng’ in Chinese), and the presence of the sesquiterpene pyridine alkaloids have immunosuppressive, antitumor-promoting and cytotoxic, antiviral, and anti-inflammatory properties.
- Sesquiterpene pyridine alkaloids are a large group of highly oxygenated sesquiterpenoids, all that based on a core C15 skeleton known as dihydro-β-agarofuran.
- **Five tripterygium pyridine alkaloids: triptolide ($C_{20}H_{24}O_6$, *m/z* 361.1646) [1], wilforgine ($C_{41}H_{47}NO_{19}$, *m/z* 858.2815) [2], wilforine ($C_{43}H_{49}NO_{18}$, *m/z* 868.3022) [3], wilfortrine ($C_{41}H_{47}NO_{20}$, *m/z* 874.2764) [4] and wilfordine ($C_{43}H_{49}NO_{19}$, *m/z* 884.2972) [5].**
- Tentative identification of these **five compounds** is based on accurate mass analysis (LC-QToF).

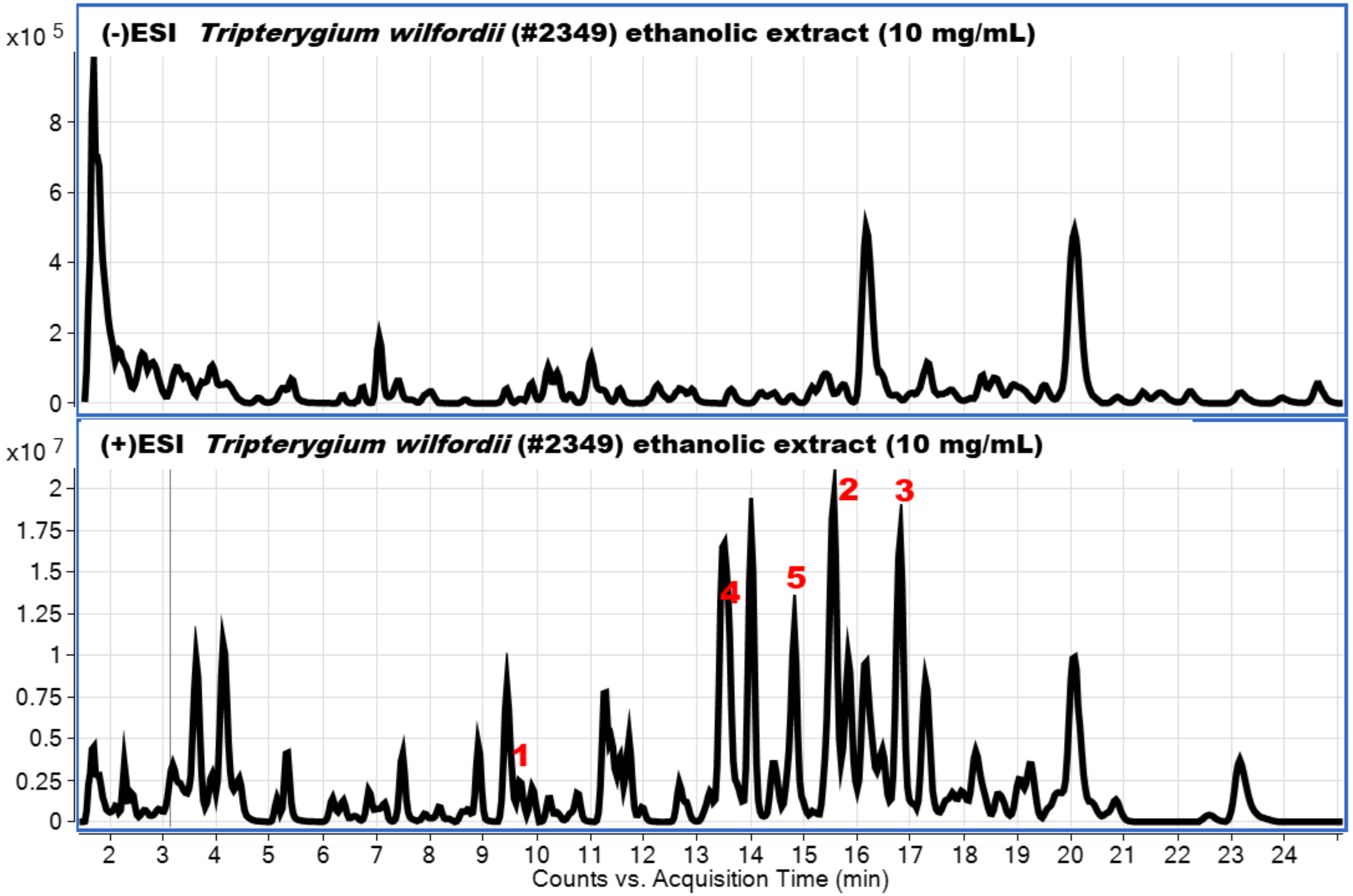

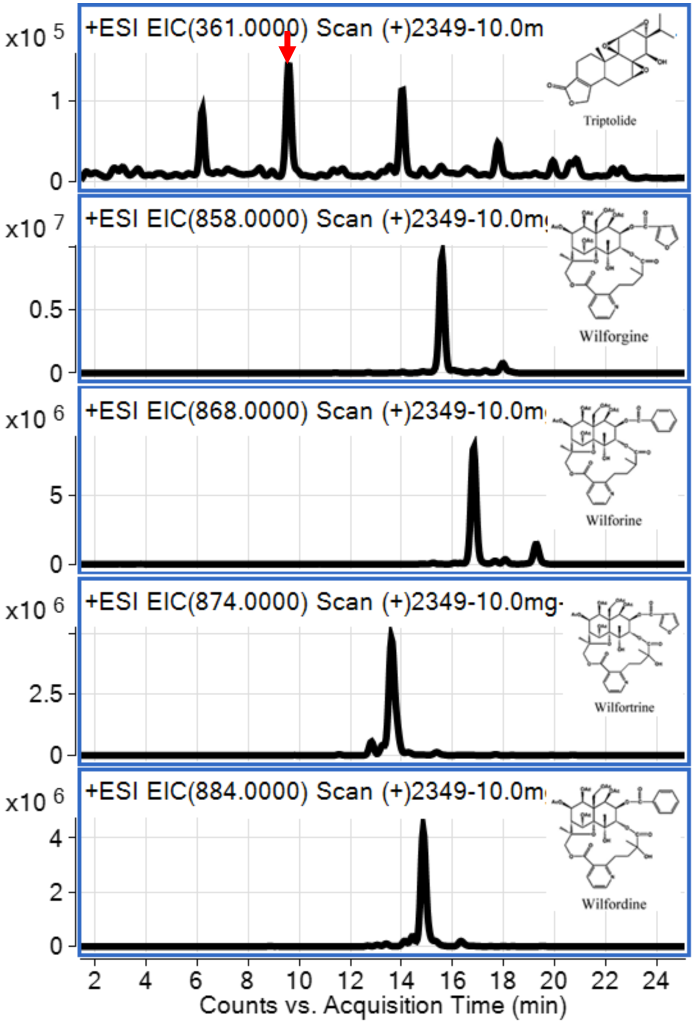

Su, M.X., Song, M., Yang, D.S., Shi, J.F., Di, B. and Hang, T.J., 2015. Simultaneous LC− MS/MS determination of five tripterygium pyridine alkaloids in dog plasma and its application to their pharmacokinetic study after oral administration of tripterygium glycosides tablets. *Journal of Chromatography B*, *990*, 31-38.
